# Orphan GPR61, GPR62 and GPR135 receptors and the melatonin MT_2_ receptor reciprocally modulate their signaling functions

**DOI:** 10.1038/s41598-017-08996-7

**Published:** 2017-08-21

**Authors:** Atsuro Oishi, Angeliki Karamitri, Romain Gerbier, Olivier Lahuna, Raise Ahmad, Ralf Jockers

**Affiliations:** 1 Inserm, U1016 Institut Cochin Paris, France; 2 CNRS UMR, 8104 Paris, France; 3 University Paris Descartes, Paris, France

## Abstract

Understanding the function of orphan G protein-coupled receptors (GPCRs), whose cognate ligand is unknown, is of major importance as GPCRs are privileged drug targets for many diseases. Recent phylogenetic studies classified three orphan receptors, GPR61, GPR62 and GPR135 among the melatonin receptor subfamily, but their capacity to bind melatonin and their biochemical functions are not well characterized yet. We show here that GPR61, GPR62 and GPR135 do not bind [^3^H]-melatonin nor 2-[^125^I]iodomelatonin and do not respond to melatonin in several signaling assays. In contrast, the three receptors show extensive spontaneous ligand-independent activities on the cAMP, inositol phosphate and ß-arrestin pathways with distinct pathway-specific profiles. Spontaneous ß-arrestin recruitment internalizes all three GPRs in the endosomal compartment. Co-expression of the melatonin binding MT_2_ receptor with GPR61, GPR62 or GPR135 has several consequences such as (i) the formation of receptor heteromers, (ii) the inhibition of melatonin-induced ß-arrestin2 recruitment to MT_2_ and (iii) the decrease of elevated cAMP levels upon melatonin stimulation in cells expressing spontaneously active GPR61 and GPR62. Collectively, these data show that GPR61, GPR62 and GPR135 are unable to bind melatonin, but show a reciprocal regulatory interaction with MT_2_ receptors.

## Introduction

G protein-coupled receptors (GPCRs) constitute the largest membrane protein family in humans that is targeted by approximately 30% of marketed drugs. More than 100 GPCRs remain orphans (without known ligand) and identifying the function of these orphan GPCRs holds great promise for future drug development^1, 2^.

Clusters of GPCRs with sequence similarity are often binding to similar types of ligands, i.e. amines, peptides or lipids^1^. This correlation has proven to be successful in deorphanizing GPCRs^1^. Recently, three orphan receptors, namely GPR61, GPR62 and GPR135, have been attributed to the melatonin receptor cluster based on a phylogenetic analysis of several mammalian species^1, 3, 4^.

The melatonin receptor subfamily has been initially defined by three members in mammals called MT_1_ and MT_2_, both binding to melatonin and the orphan GPR50, which does not bind to melatonin^5, 6^. Typical signal transduction pathways involve inhibition of cAMP production by MT_1_ and MT_2_ and stimulation of inositol phosphate production by MT_1_. GPR50 seems to be silent on its own but exhibits a profound inhibitory effect on MT_1_ function in MT_1_/GPR50 heteromeric complexes, revealing an alternative, ligand-independent action mode of orphan GPCRs^7^.

For GPR61, GPR62 and GPR135, currently available information is much more restricted. Evolutionary conservation of the three receptors in fish, amphibians, reptiles, birds, and mammals underlines their importance (http://www.ncbi.nlm.nih.gov/gene/?term=GPR61, or GPR62 or GPR135). They are abundantly expressed in the brain and in some other tissues including pancreatic beta-cells^8–11^. GPR61-deficient mice are obese and hyperphagic^12^. The phenotype of GPR62 and GPR135 knockout mice was not reported yet. Analysis of the GPR61 promoter in discordant monozygotic twins showed that hypermethylation of this region is associated with type 2 diabetes (T2D)^13^. Cold exposure increased GPR135 expression in mouse liver but not in adipose tissue^14^. In an epigenetic study GPR135 was identified as one of the methylated placental genes associated with maternal cigarette smoking during pregnancy^15^, a condition known to increase the risk for miscarriage, low birth weight, preterm birth and obesity^16^. Collectively, these data point to a possible role in energy metabolism related functions. In terms of intracellular signaling, conflicting results have been reported on the capacity of GPR61 to constitutively activate either G_s_ or G_i_ protein pathways^17, 18^. For GPR62 constitutive activation of the G_i_/cAMP pathway has been suggested^18^. Whether these receptors bind melatonin or any other ligand has not been addressed.

We report here the first side-by-side functional analysis of GPR61, GPR62 and GPR135 in terms of their capacity to activate intracellular signaling pathways in a spontaneous and melatonin-dependent manner. In addition, we show that the GPRs affect MT_2_ signaling, most likely by an allosteric mechanism operating in receptor heteromers and that inversely, MT_2_ activation impacts on the constitutive activity of GPR61 and GPR62 on the cAMP pathway through signaling cross-talk.

## Result

### Expression and 2-[^125^I]-MLT and [^3^H]-MLT binding capacity of GPR61, GPR62 and GPR135 in HEK293T cells

**Figure 1:**
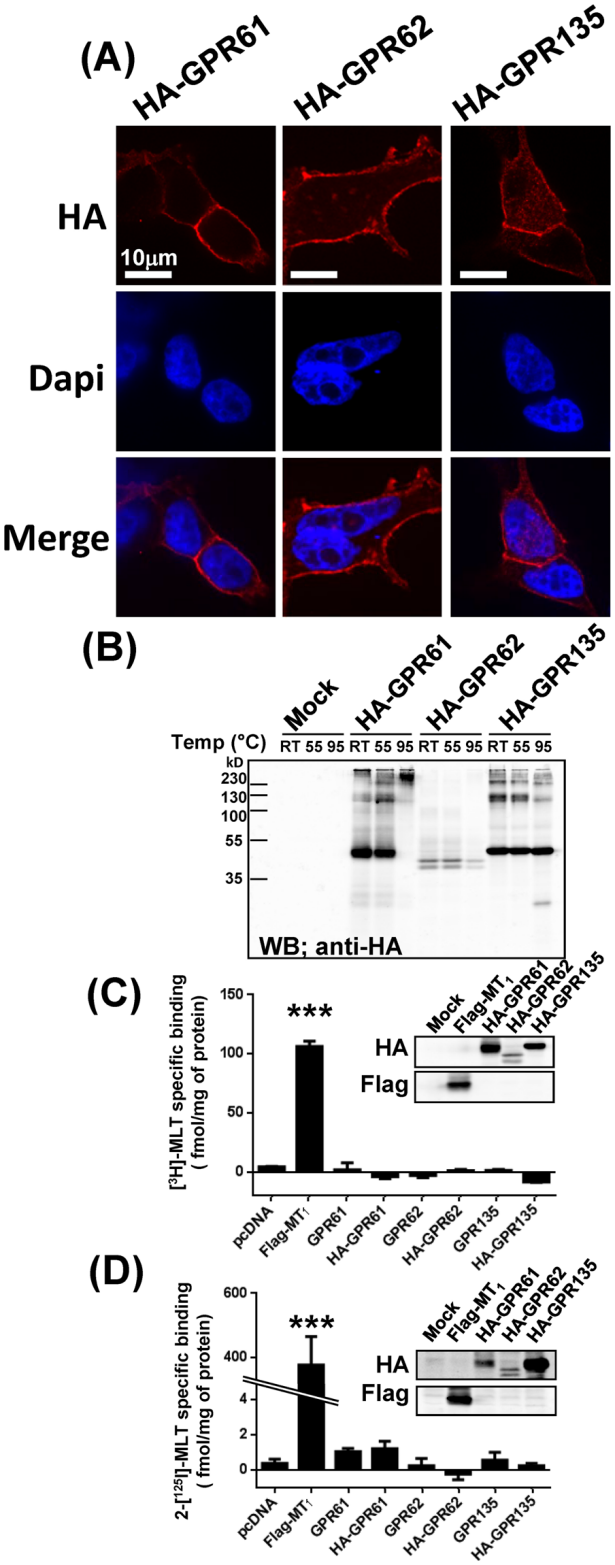
Expression and [^3^H]-MLT and 2-[^125^I]-MLT binding to HA-GPR61, HA-GPR62 and HA-GPR135 expressed in HEK293T cells. (**A**) The expression of HA-tagged (N-terminal) GPR61, GPR62 and GPR135 at the cell surface of intact HEK293T cells was detected by confocal microscopy with anti-HA antibodies. (**B**) Expression of HA-GPR61, HA-GPR62 and HA-GPR135 in crude membranes prepared from HEK293T cells was monitored by western blot. Different denaturating conditions were applied: RT; room temperature overnight, 55 °C 15 min, or 95 °C 10 min, all in the presence of DTT. (**C**,**D**) Binding of [^3^H]-MLT (**C**) and 2-[^125^I]-MLT (**D**) to crude membrane prepared from HEK293 cells expressing HA-tagged or untagged GPR61, GPR62 or GPR135. Expression of HA-tagged receptors was verified by western blot (insets). Flag-MT_1_ was used as positive control. Representative results are shown for panels A and B; similar results were obtained in three additional experiments. Data in panels C and D represent the mean ± SEM of three independent experiments performed in duplicates. ***P < 0.001.

Since no validated antibodies are available for GPR61, GPR62 and GPR135, we first generated epitope tagged version of these receptors by adding an HA-tag at their amino-terminal extremity. Successful expression of all three receptors at the cell surface of intact HEK293T cells was shown by confocal immunofluorescence microscopy (Fig. 1A) and by western blot in crude membrane preparations (Fig. 1B). When samples were denatured at standard conditions (10 min, 95 °C) before separation by SDS-PAGE, only HA-GPR135 was detected at the predicted size of the receptor monomer, HA-GPR62 was undetectable and HA-GPR61 migrated at a very high molecular weight, most likely corresponding to receptor aggregates. When denatured at 55 °C for 15 min or room temperature for 16 hours, HA-GPR61 and HA-GPR62 were readily detected at the expected molecular weight of 40–50 kDa (Fig. 1B) confirming successful expression of all three receptors.

**Figure 2:**
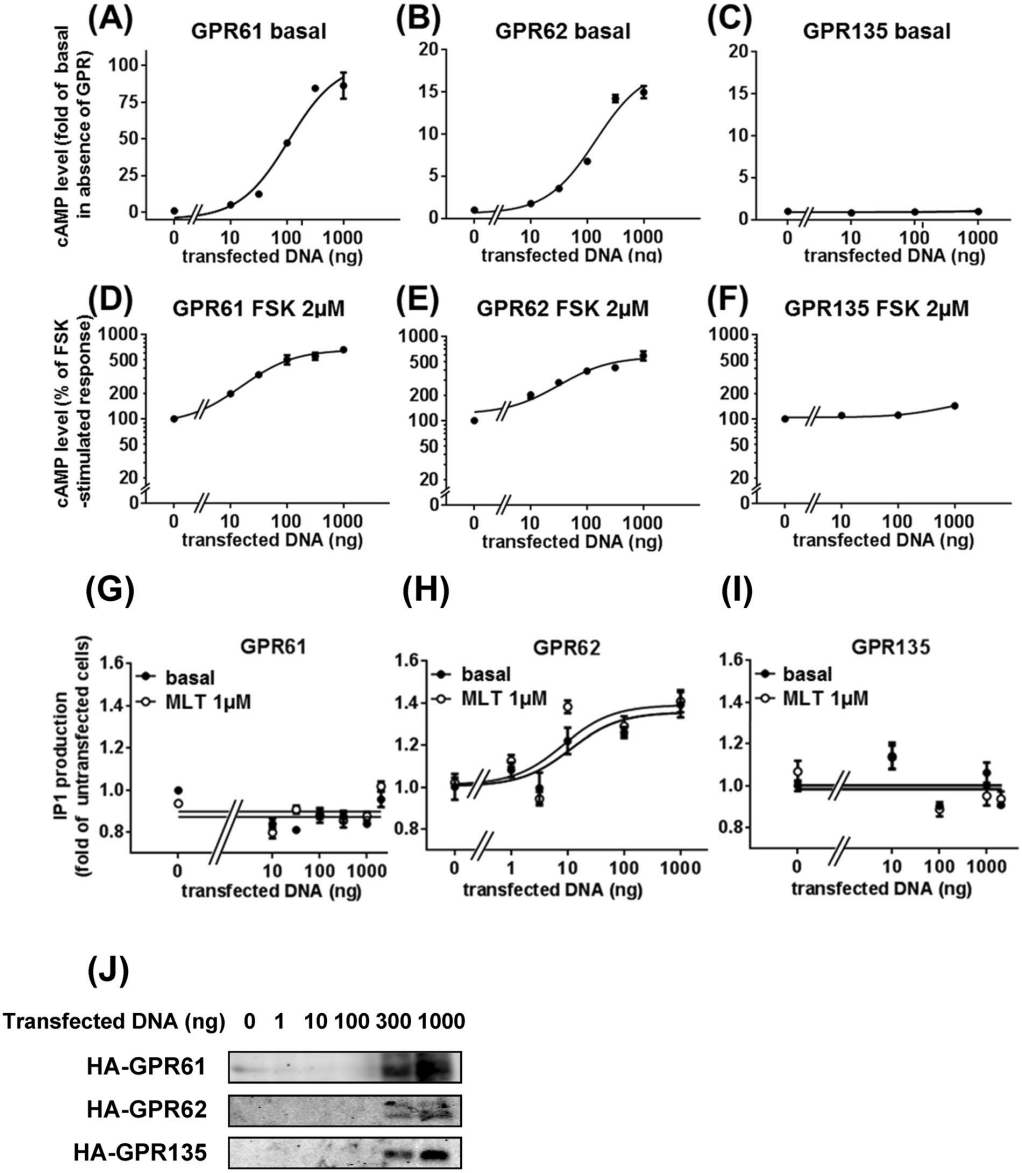
Effect of GPR61, GPR62 and GPR135 expression on cAMP and inositol phosphate production in HEK293T cells. Basal (**A–C**) and forskolin-stimulated (**D–F**) cAMP accumulation in HEK293 cells as a function of transfected vector DNA expressing HA-GPR61, HA-GPR62 and HA-GPR135. Inositol phosphate (IP1) production was determined in HEK293T cells transfected with different quantities of HA-GPR61 (**G**), HA-GPR62 (**H**) and HA-GPR135 (**I**) expression vectors in the absence or presence of 1 µM melatonin (MLT). (**J**) Representative western blot of HA-GPR61, HA-GPR62 or HA-GPR135 expression control of experiments shown in panels A–I. Data are expressed as mean ± SEM. from three independent experiments performed in triplicates. ***P < 0.0001.

We next examined the capacity of HA-GPR61, HA-GPR62 and HA-GPR135 to bind the two commercially available melatonin receptor radioligands [^3^H]-MLT (Fig. 1C) and 2-[^125^I]-MLT (Fig. 1D). Both radioligands were used at saturating concentrations for MT_1_ and MT_2_ receptors (1 nM and 300 pM for [^3^H]-MLT and 2-[^125^I]-MLT, respectively). Non-specific binding was determined in the presence of an excess (10 µM) of the corresponding unlabeled ligands. Whereas both radioligands showed specific binding for the positive control MT_1_ receptor, no specific binding was observed for HA-GPR61, HA-GPR62 and HA-GPR135, despite successful expression as monitored in parallel by western blot. To exclude any potential interference of the HA tag, the untagged versions of the three receptors were also included in the binding assay (Fig. 1C,D). However, no specific binding was observed either. Similar negative results were obtained at a higher (2 nM) concentration of 2-[^125^I]-MLT (not shown). These data indicate that GPR61, GPR62 and GPR135 do not bind melatonin or iodomelatonin at concentration up to 2 nM.

### GPR61 and GPR62 show constitutive activity on the G_s_/cAMP pathway but not on the G_i/o_/cAMP pathway

We next examined the capacity of HA-tagged GPR61, GPR62 and GPR135 to promote cAMP production in HEK293T cells. Expression of increasing amounts of GPR61 and GPR62 increased cAMP levels (Fig. 2A,B,J). Similar results were obtained with untagged receptors or when cells expressing the receptors were starved overnight, excluding serum-derived potential ligands (Supplementary Fig. S1). No effect was observed in cells expressing GPR135 (Fig. 2C,J). These data suggest that GPR61 and GPR62 are constitutively active on the G_s_/cAMP pathway and that this activity is unlikely to be due to the presence of ligands for GPR61 and GPR62 in the serum.

We then examined the effect of GPR61, GPR62 and GPR135 expression on forskolin-induced cAMP accumulation to investigate the possibility of constitutive activity on the G_i/o_ pathway. In contrast to a previous report^18^, expression of GPR61 and GPR62 did not decrease but rather amplified forskolin-induced cAMP accumulation (Fig. 2D,E), whereas GPR135 expression had no effect (Fig. 2F). The half maximal effect of the transfected DNA amount was very similar in the presence and absence of forskolin for GPR61 (pEC_50_ = 7.12 ± 0.14 (Fig. 2D) and 7.44 ± 0.14 g (Fig. 2A) of DNA vector) and GPR62 (pEC_50_ = 6.83 ± 0.068 (Fig. 2E) and 7.18 ± 0.15 g (Fig. 2B) of DNA vector). Treatment of cells with melatonin (1 µM) did not affect cAMP levels neither in the absence (Supplementary Fig. S2A) nor presence of forskolin (different concentrations) (Supplementary Fig. S2C–E), but decreased basal cAMP levels in cells expressing MT_2_, as expected (Supplementary Fig. S2B). These data show that GPR61 and GPR62 spontaneously increase basal and forskolin-stimulated cAMP levels, whereas GPR135 is silent on this pathway.

### GPR62 shows constitutive activity on the G_q_/IP1 pathway

We then investigated the capacity of increasing expression levels of HA-tagged GPR61, GPR62 and GPR135 to promote inositol phosphate (IP1) production in HEK293T cells (Fig. 2G–J). IP1 production was increased in a dose-dependent manner up to 40% in cells expressing HA-GPR62 (Fig. 2H,J). The magnitude of this effect was comparable to that induced by a saturating concentration of angiotensin II (AngII) (approximately 100%) in cells expressing the angiotensin II type I receptor (AT1R) (Supplementary Fig. S2F). Incubation of HA-GPR62 expressing cells with melatonin had no additional effect. IP1 levels remained unchanged in cells expressing GPR61 and GPR135 in the absence and presence of melatonin (Fig. 2G,I). These data indicate that GPR62 (but not GPR61 and GPR135) is constitutively active on the G_q_/IP1 pathway in HEK293T cells.

### GPR61, GPR62 and GPR135 show spontaneous ß-arrestin recruitment

**Figure 3:**
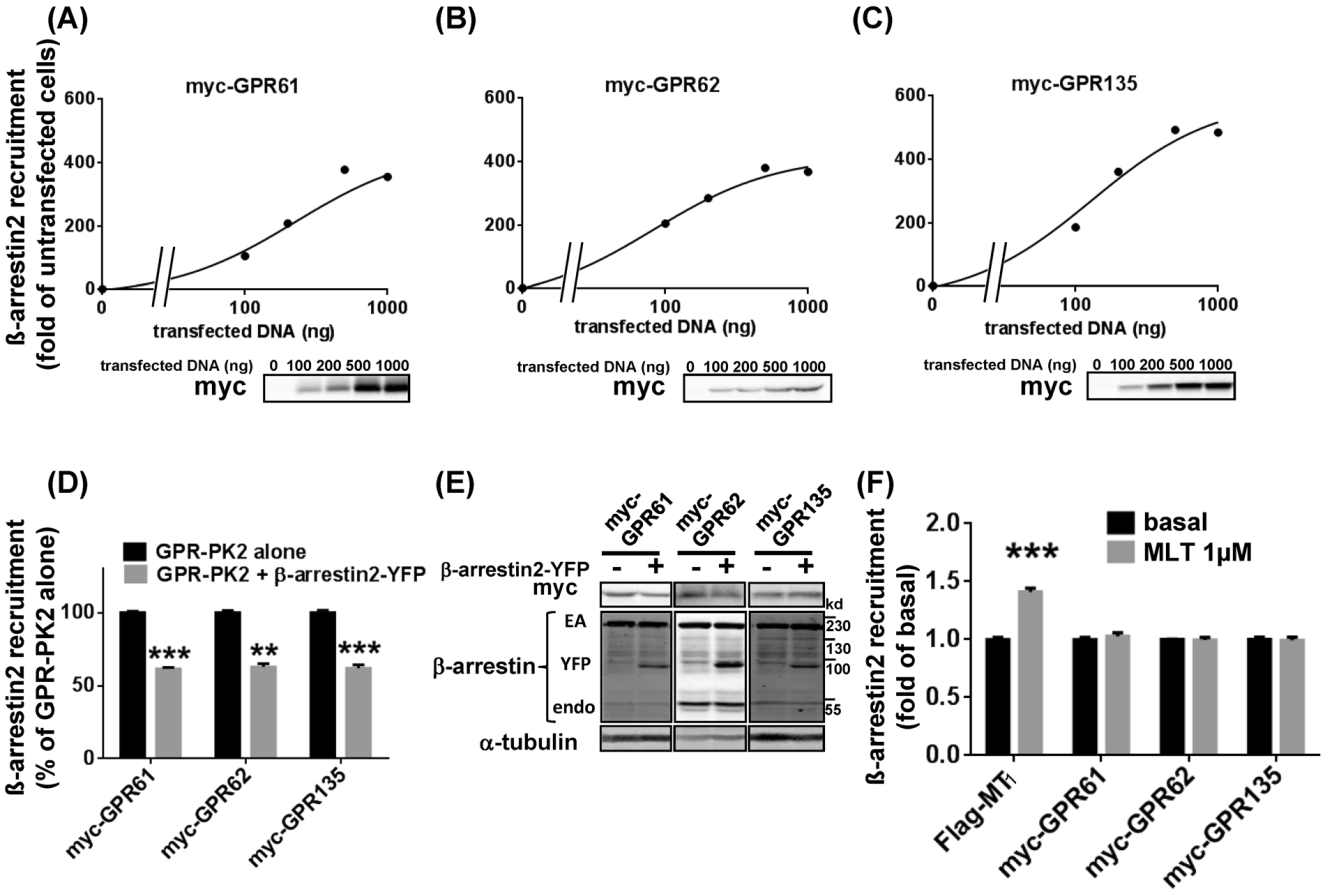
Agonist-independent association between β-arrestin2 and GPR61, GPR62 and GPR135. (**A–C**) ß-arrestin recruitment was determined in HEK-arrestin2-EA cells stably expressing the ß-arrestin2-EA fusion protein and transfected with different quantities of myc-GPR61-PK2 (**A**), myc-GPR62-PK2 (**B**) or myc-GPR135-PK2 (**C**) expression vectors. Expression levels were monitored by western blot. (**D**,**E**) ß-arrestin2 competition assay. HEK-arrestin-EA cells were transfected with 500 ng of GPR-PK2 vectors with or without an additional 500 ng of ß-arrestin2-YFP vector (**D**). Expression levels of myc-GPR61, myc-GPR62 and myc-GPR135 and ß-arrestins were monitored by western blot (**E**). Effect of melatonin (1 µM) on ß-arrestin2 recruitment to GPR61, GPR62 and GPR135 (1 ug) (**F**). Flag-MT_1_-PK2 was used as a positive control. Western blots shown are representative and have been performed for each experiment. Data are expressed as mean ± SEM from 3 independent experiments performed in triplicates. **P < 0.01, ***P < 0.001.

To evaluate the capacities of GPR61, GPR62 and GPR135 to signal through G protein-independent pathways, we investigated ß-arrestin recruitment with the PathHunter™ enzyme complementation assay. Expression of increasing amounts of GPR61, GPR62 and GPR135 containing an N-terminal myc tag and a C-terminal PK2 tag (to monitor recruitment of the ß-arrestin2-EA (enzyme activator)) dose-dependently recruited ß-arrestin2-EA for all three receptors in HEK293 cells stably expressing ß-arrestin2-EA (Fig. 3A–C). To confirm the specificity of this constitutive recruitment, we performed a ß-arrestin2 competition assay by additional transfection of ß-arrestin2-YFP. We observed a 40–50% reduction in ß-arrestin2-EA recruitment (Fig. 3D) without any change in the expression levels of the receptors, ß-arrestin2-EA nor endogenous ß-arrestins1/2 (Fig. 3E). Stimulation with melatonin (1 µM) increased ß-arrestin2-EA recruitment in cells expressing Flag-MT_1_-PK2, as expected, but had no effect on the constitutive ß-arrestin2-EA recruitment in cells expressing GPR61, GPR62 and GPR135 (Fig. 3F). Similar observations were made in HEK293 cells stably expressing ß-arrestin1-EA. All three GPRs constitutively associated with ß-arrestin1 (Supplementary Fig. S3A), which was not further modulated by melatonin (Supplementary Fig. S3B).

To evaluate the physiological significance of spontaneous ß-arrestin recruitment to orphan receptors, we chose GPR135, which was reported to be expressed in human pancreatic beta cells^10^. We first determined the minimal amount (1 ng) of GPR135-PK2 expression vector able to promote significant ß-arrestin2 recruitment in HEK293 cells (Supplementary Fig. S4A). Q-PCR experiments showed then that GPR135 mRNA levels measured at these conditions closely matched those observed in the human pancreatic EndoC-βH1 cell line^19^ (Supplementary Fig. S3B). Hence, in terms of its agonist-independent activity, GPR135 can be considered a ß-arrestin-biased receptor at mRNA levels similar to those observed in pancreatic beta cells. Taken together, the data on ß -arrestin recruitment indicate that GPR61, GPR62 and GPR135 associate with ß-arrestin1 and 2 in a spontaneous and agonist-independent manner.

### GPR61, GPR62 and GPR135 co-localized with ß-arrestin2 in the endosome

**Figure 4:**
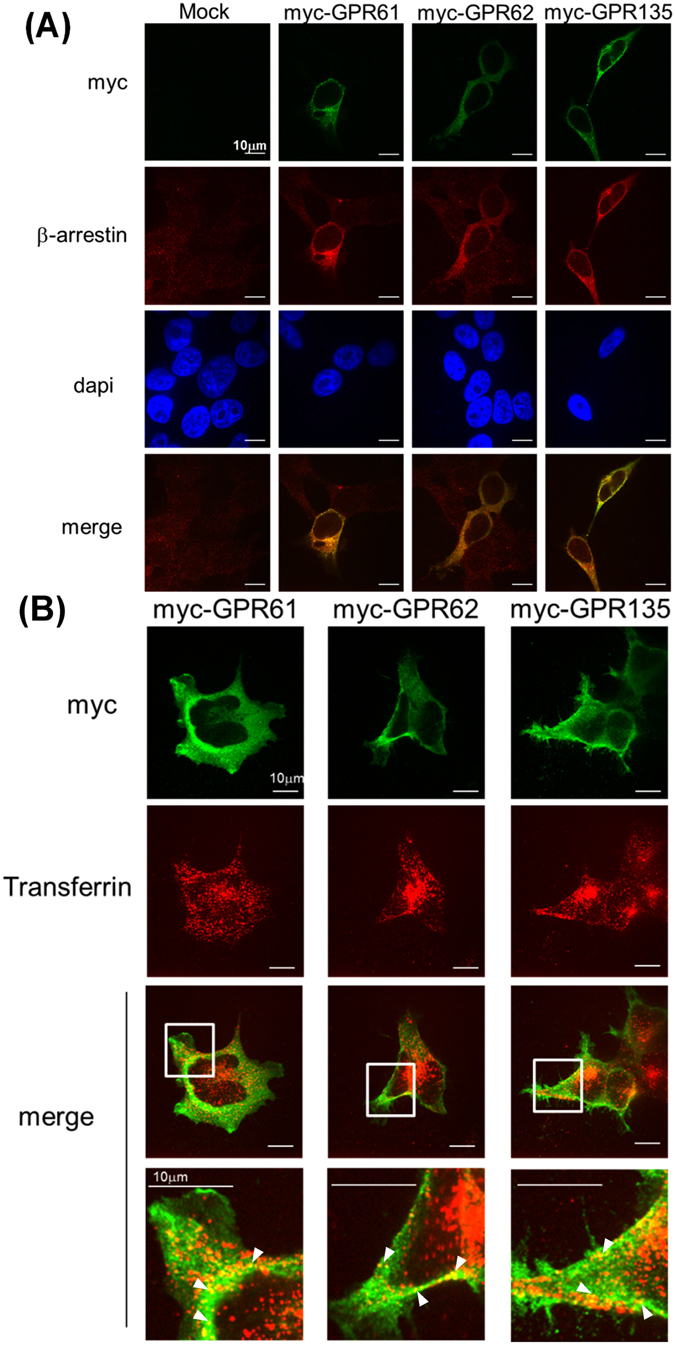
Subcellular localization of myc-GPR61, myc-GPR62, myc-GPR135. (**A**,**B**) Myc-tagged GPR61, GPR62 or GPR135 were expressed in HEK-arrestin2-EA cells. (**A**) The subcellular localization of GPRs and ß-arrestins was detected in permeabilized cells by confocal microscopy with anti-myc and anti-ß-arrestin antibodies. (**B**) Cells were incubated with Transferrin-Alexa555 (5 μg/ml for 30 minuites in 37 °C) to label endosomal compartments and permeabilized. GPRs were detected with anti-myc antibodies and colocalization with internalized Transferrin-Alexa555 was monitored by confocal fluorescence microcopy.

To confirm the association between GPR61, GPR62, GPR135 and ß-arrestin2, we studied the localization of ß-arrestin2 and GPRs by confocal microscopy in permeabilized ß-arrestin2-EA HEK293 cells. Expression of GPRs dramatically changed the ß-arrestin2 staining from a diffuse staining in the absence of GPRs to punctuate intracellular staining in the presence of GPRs (Fig. 4A). Within these intracellular compartments, GPRs colocalized with ß-arrestin2 (Fig. 4A) thus confirming the constitutive recruitment of ß-arrestin2 by GPRs detected in the PathHunter™ enzyme complementation assay. Internalization of GPRs into endosomes was further confirmed by their colocalization with internalized, fluorescently labeled transferrin (Fig. 4B). Taken together, GPRs constitutively internalize and colocalize at steady-state with ß-arrestins in the endosomal compartment.

### GPR61, GPR62 and GPR135 inhibit melatonin induced ß-arrestin recruitment of MT_2_ but not MT_1_

**Figure 5:**
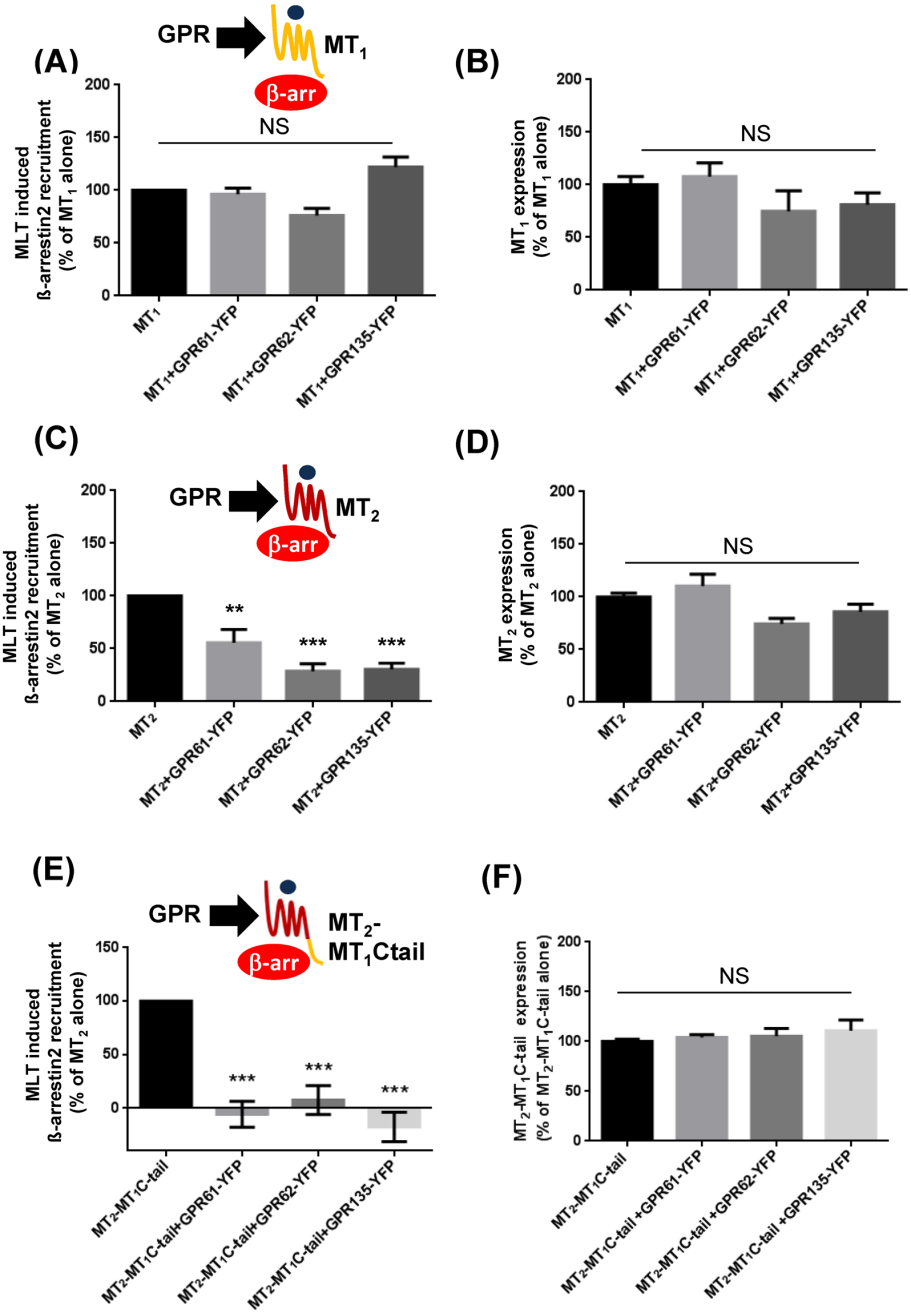
Effect of GPR61, GPR62 and GPR135 expression on melatonin-induced β-arrestin2 recruitment to MT_1_ and MT_2_. (**A**,**C**,**E**) ß-arrestin recruitment was determined in HEK-arrestin-EA cells expressing the ß-arrestin2-EA fusion protein and the indicated receptors upon treatment with melatonin (1 µM). Results are expressed as % of melatonin response obtained in cells expressing Flag-MT_1_-PK2 (**A**), HA-MT_2_-PK2 (**C**) or myc-MT_2_-MT_1_-Ctail-PK2 (**E**) alone. Expression levels of Flag-MT_1_-PK2 (**B**), HA-MT_2_-PK2 (**D**) and chimera myc-MT_2_-MT_1_-Ctail-PK2 (**F**) were monitored by ELISA assay. Data are expressed as mean ± SEM from at least 3 independent experiments. **P < 0.01, ***P < 0.001.

To investigate whether spontaneous ß-arrestin recruitment to GPR61, GPR62 or GPR135 modulates melatonin MT_1_ and MT_2_ receptor signaling, we expressed MT_1_-PK2 or MT_2_-PK2 in the presence of GPR61-YFP, GPR62-YFP or GPR135-YFP. Whereas melatonin induced ß-arrestin2 recruitment to MT_1_ was unaffected (Fig. 5A), recruitment to MT_2_ was significantly inhibited by all three orphan receptors (Fig. 5C) under conditions of constant receptor expression levels (Fig. 5B,D, Supplementary Fig. S5A). The melatonin receptor antagonist S20928 was unable to promote ß-arrestin2 recruitment to MT_1_ and MT_2_- irrespective of the absence or presence of the three orphan receptors (Supplementary Fig. S5B,C). These data show that GPR61, GPR62 and GPR135 inhibit melatonin-induced ß-arrestin2 recruitment specifically to MT_2_.

To better understand this differential behavior of MT_1_ and MT_2_, we designed a MT_2_ chimera containing the Ctail of MT_1_. The Ctail of GPCRs is considered to be the major molecular determinant involved in the first phase of ß-arrestin recruitment^20^. All three GPRs inhibited ß-arrestin2 recruitment to the MT_2_-MT_1_C-tail chimera (Fig. 5E,F) indicating that differences in the C-tails don’t contribute to the differential phenotype of MT_1_ and MT_2_.

To further study the specificity of the inhibitory effect of GPRs on ß-arrestin recruitment to MT_2_, we examined the effect of GPR61, GPR62 or GPR135 expression on the capacity of AT1R to activate the ERK, a pathway known to be ß-arrestin dependent in HEK293 cells^21^. Neither ERK activation by the natural angiotensin II agonist nor with the ß-arrestin biased [Sal^1^-Ile^4^-Ile^8^]-AngII (SII) agonist was modified by the expression of GPRs (Supplementary Fig. S5D). Taken together, GPR61, GPR62 and GPR135 are unlikely to be general regulators of the ß-arrestin function by sequestering ß-arrestin away from other GPCRs.

### GPR61, GPR62 and GPR135 form heteromers with MT_2_

**Figure 6:**
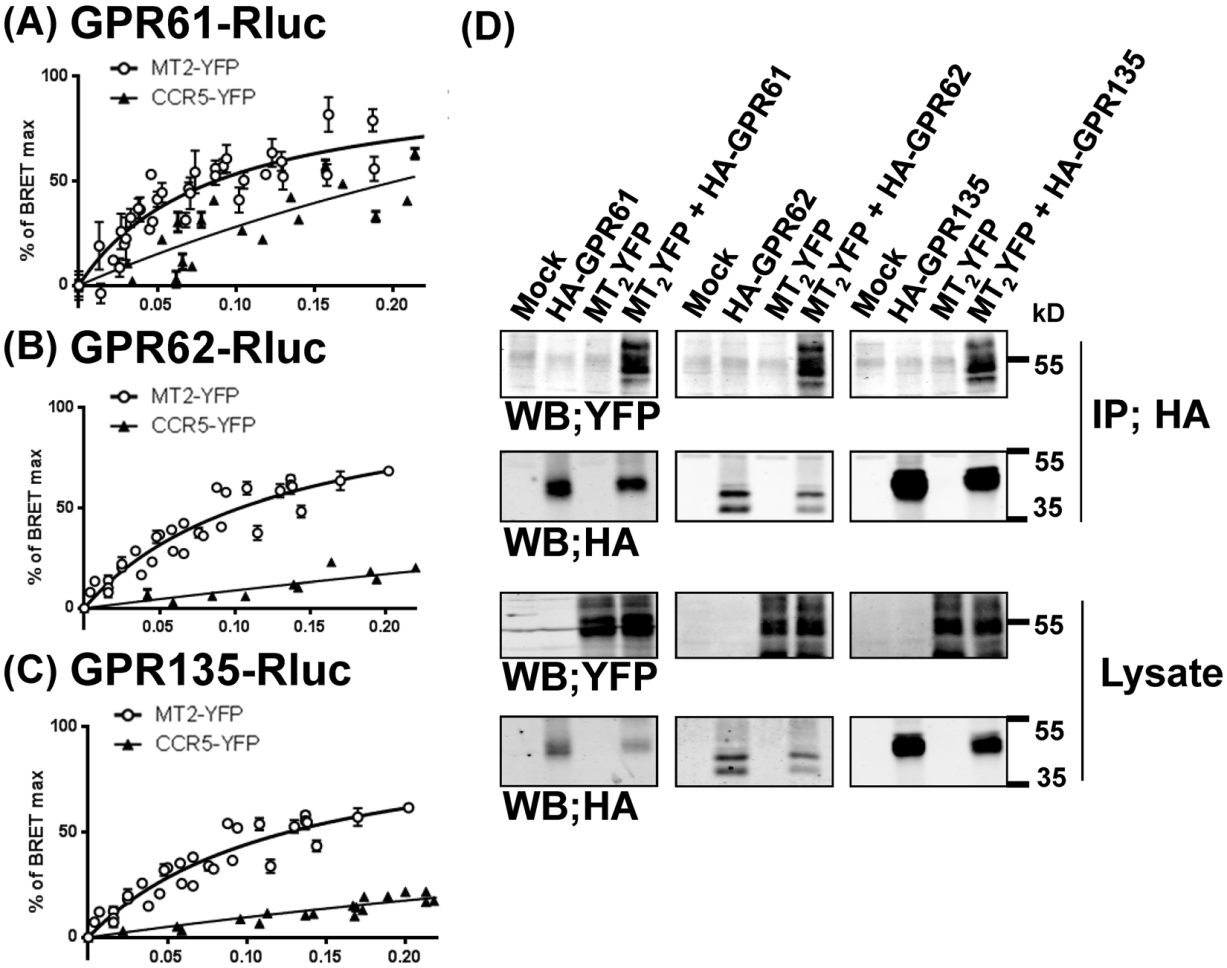
Heteromer formation of MT_2_ with GPR61, GPR62 and GPR135 in living HEK 293 T cells. (**A–C**) BRET donor saturation curves were generated by expressing constant amounts of GPR61-Rluc (**A**), GPR62-Rluc (**B**) and GPR135-Rluc (**C**) fusion proteins (energy donor) and increasing quantities of the indicated YFP-tagged receptors (energy acceptor) in HEK293T cells. The curves represent at least 3 individual saturation curves. Curves were normalized to BRET_max_ values. Curves obtained for the BRET acceptors MT_2_-YFP were best fitted with a nonlinear regression equation assuming a single binding site, those obtained for CCR5-YFP were best fitted with a linear regression equation. (**D**) Co-immunoprecipitation of MT_2_/GPR61 (left), MT_2_/GPR62 (middle) and MT_2_/GPR135 (right) heteromers. Lysates from HEK293T cells expressing the indicated receptors were immunoprecipitated (IP) with anti-HA antibodies, and the presence of co-precipitated MT_2_-YFP was detected with anti-GFP antibodies (upper Western blot (WB)). Expression of fusion proteins in lysates was assessed by immunoblotting with the indicated antibodies (lower Western blots). Data are representative of three experiments.

To better understand the mechanism of the inhibitory effect of the three orphan receptors on ß-arrestin2 recruitment to MT_2_, we studied the capacity of MT_2_ to engage into heteromeric complexes with the three orphan receptors by Bioluminescence Resonance Energy Transfer (BRET) in living HEK293T cells. Donor saturation curves with specific interactions were obtained for all three combinations (GPR61/MT_2_, GPR62/MT_2_, GPR135/MT_2_) whereas BRET with the negative control CCR5 receptor increased linearly, indicating a non-saturatable, non-specific interaction (Fig. 6A–C). This observation was further confirmed by co-immunoprecipitation experiments, detecting heteromer formation in cells co-expressing MT_2_ with one of the orphan receptors (Fig. 6D).

We then wanted to explore whether heteromer formation also modulates the inhibitory effect of MT_2_ on forskolin-stimulated cAMP production. Melatonin concentration- response curves showed robust cAMP inhibition with pEC_50_ values that were not affected by the presence of GPR61, GPR62 or GPR135 indicating that the inhibitory allosteric effect of the three orphan receptors on MT_2_ function is pathway specific (Supplementary Fig. S6A–E). Similar results were obtained for MT_1_ expressing cells, in which GPR expression did not affect forskolin-stimulated cAMP production (Supplementary Fig. S7A–E). Collectively, our data indicate that the three orphan GPRs engage into heteromers with MT_2_, which possibly modifies the capacity of MT_2_ to specifically recruit ß-arrestin2 but not to inhibit cAMP production.

### Effect of MT_2_ stimulation by melatonin on the constitutive activity of GPR61, GPR62 and GPR135

**Figure 7:**
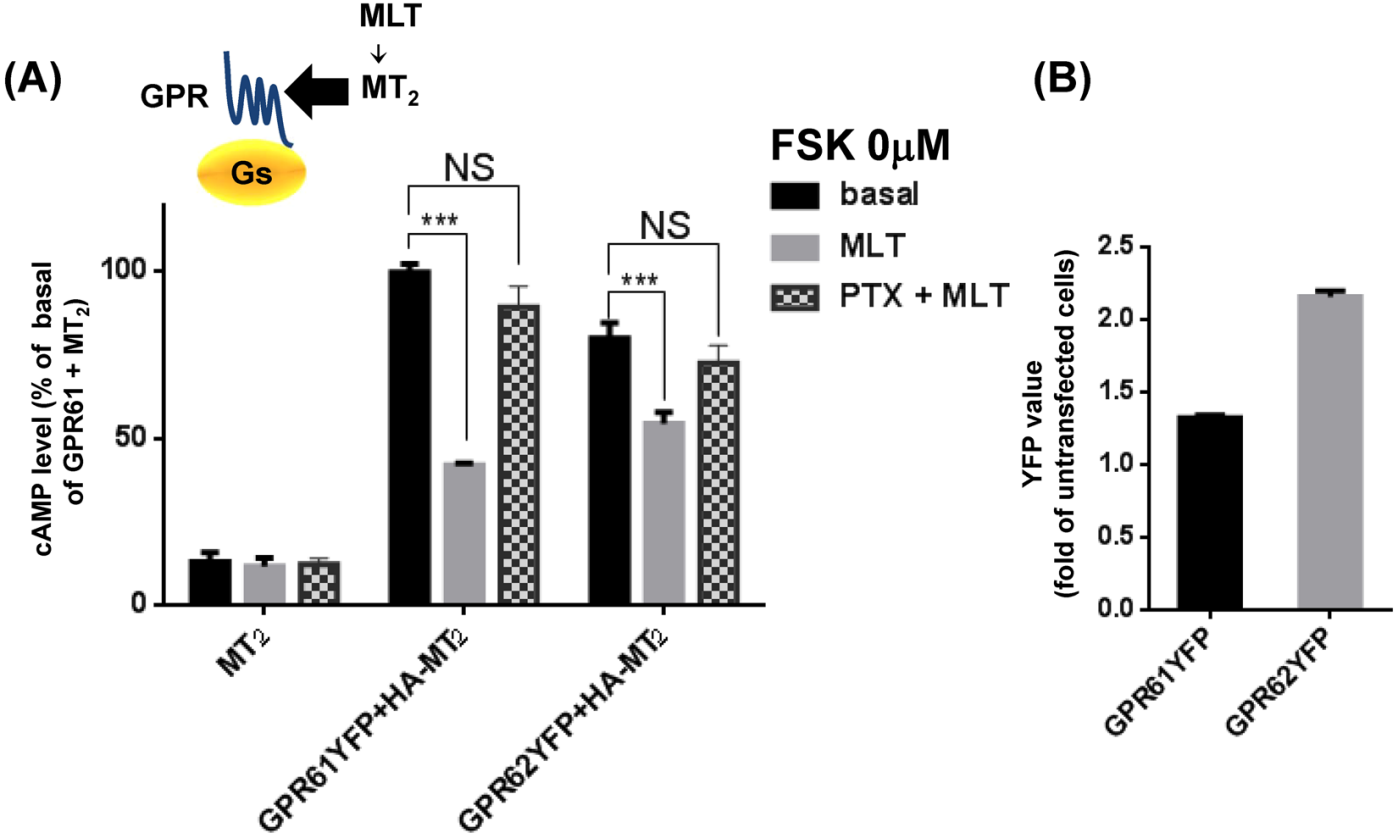
Inhibition of constitutive G_s_/cAMP signaling of GPR61 and GPR62 by MT_2_. (**A**) HA-MT_2_ was expressed in HEK293T cells alone or together with GPR61-YFP or GPR62-YFP and cAMP levels determined in untreated and melatonin-treated (1 µM, 30 minutes) cells. Some cells were pre-treated with pertussis toxin (200 ng/ml × 4 hr) pretreatment. (**B**) Expression levels of GPR61YFP and GPR62YFP were determined in parallel by measuring YFP fluorescence. Data are expressed as mean ± SEM from at least 3 independent experiments.

We next examined the opposite configuration, namely whether MT_2_ activation affects the constitutive activity of the three GPRs. Neither the constitutive activity of GPR62 on the inositol phosphate pathway (Supplementary Fig. S8), nor agonist independent ß-arrestin recruitment of GPR61, GPR62 or GPR135 (Supplementary Fig. S9) was affected by melatonin-induced activation of MT_2_. In contrast, MT_2_ activation readily diminished elevated cAMP levels in cells expressing GPR61 or GPR62 (Fig. 7A,B). Pretreatment of cells with pertussis toxin, to inactivate G_i/o_ proteins, prevented this effect (Fig. 7A). Collectively, these data suggest that the allosteric regulation between MT_2_ and GPR protomers is unidirectional (from GPRs to MT_2_) as no evidence for allosteric regulation of spontaneous GPR function by MT_2_ was observed. In contrast G_i/o_ activation by MT_2_ is able to counteract the constitutive activity of GPR61 and GPR62 on the G_s_/cAMP pathway through G_i/o_-dependent signaling cross-talk.

## Discussion

Here we reported the first extensive and side-by-side characterization of three orphan GPCRs, GPR61, GPR62 and GPR135, previously proposed to belong to the melatonin receptor subfamily. We show by using binding and functional assays that melatonin is not the cognate ligand for GPR61, GPR62 and GPR135, which remain orphans. In contrast, all three orphan receptors display spontaneous receptor activity on various signaling pathways. The most distinct profile is observed for GPR135 which shows only spontaneous activity for ß-arrestin recruitment and can thus be considered ß-arrestin-biased as compared to G_s_-, G_i_- and G_q_-dependent pathways. The constitutive activity observed in HEK293 cells, in particular on the G_s_ and the ß-arrestin pathways, could be very useful to setup screening assays for inverse agonists for these orphan receptors. In addition, GPR61, GPR62 and GPR135 are able to modulate melatonin promoted functions of the MT_2_ receptor most likely by forming heteromeric complexes. This effect seems to be signaling pathway-specific as only seen for ß-arrestin2 recruitment but not for inhibition of cAMP production. Inversely, MT_2_ stimulation modulates specifically the constitutive activation of the cAMP pathway by GPR61 and GPR62 revealing all together a reciprocal regulatory signaling network between MT_2_ and GPR61, GPR62 and GPR135.

GPR61, GPR62 and GPR135 are three out of approximately 100 remaining orphan GPCRs. Despite their recent attribution to the melatonin receptor cluster^1, 3, 4^, we didn’t find any supporting evidence that melatonin is directly binding to and activating GPR61, GPR62 and GPR135 as monitored in [^3^H]-MLT and 2-[^125^I]-MLT binding assays and several functional assays determining cAMP, IP1 levels and ß-arrestin2 recruitment. The case of GPR50 illustrates the possibility of alternative functional interactions between melatonin signaling and orphan receptors that affects melatonin-induced signaling through heteromerization with MT_1_^7, 22, 23^. Indeed, a similar, although not identical, scenario seems to exist for GPR61, GPR62 and GPR135, which engage into heteromers with MT_2_ and inhibit melatonin-induced ß-arrestin recruitment to MT_2_. In contrast to GPR50, which leads to a general loss of MT_1_ function in terms of G protein signaling and ß-arrestin recruitment most likely due to sterical hindrance between the large GPR50 Ctail and signaling molecules, GPR61, GPR62 and GPR135 affect only ß-arrestin recruitment but not G_i/o_ protein activation by MT_2_. Recently Lefkowitz and colleagues showed that the interaction of ß-arrestin and GPCR is biphasic, the 1st phase consists of the docking of the N-domain of ß-arrestin to the C-terminal tail of GPCRs and the 2nd phase in the binding of the finger-loop region of ß-arrestin to the transmembrane core of GPCRs^20, 24, 25^. Our result that the Ctail of MT_2_ (1st phase) is not responsible for the observed differential regulation between MT_1_ and MT_2_ suggested that a negative allosteric effect of the three orphan GPCRs is due to the ability of the transmembrane core of MT_2_ to reach the ß-arrestin-competent conformation (2nd phase) in the heteromer. Along this line, the absence of effect of the three orphan receptors on MT_2_ promoted G protein-dependent signaling could be explained by the fact that the G protein- and the ß-arrestin-competent receptor conformations have been shown to be distinct and to be differentially stabilized by biased ligands^26–29^. The identification of cognate or surrogate ligands for GPR61, GPR62 and GPR135 would be of interest in this respect as they are expected to modulate the receptor conformation which is likely to have an impact on the allosteric regulation within the heteromer, as already shown for several other heteromers^30–33^.

**Figure 8:**
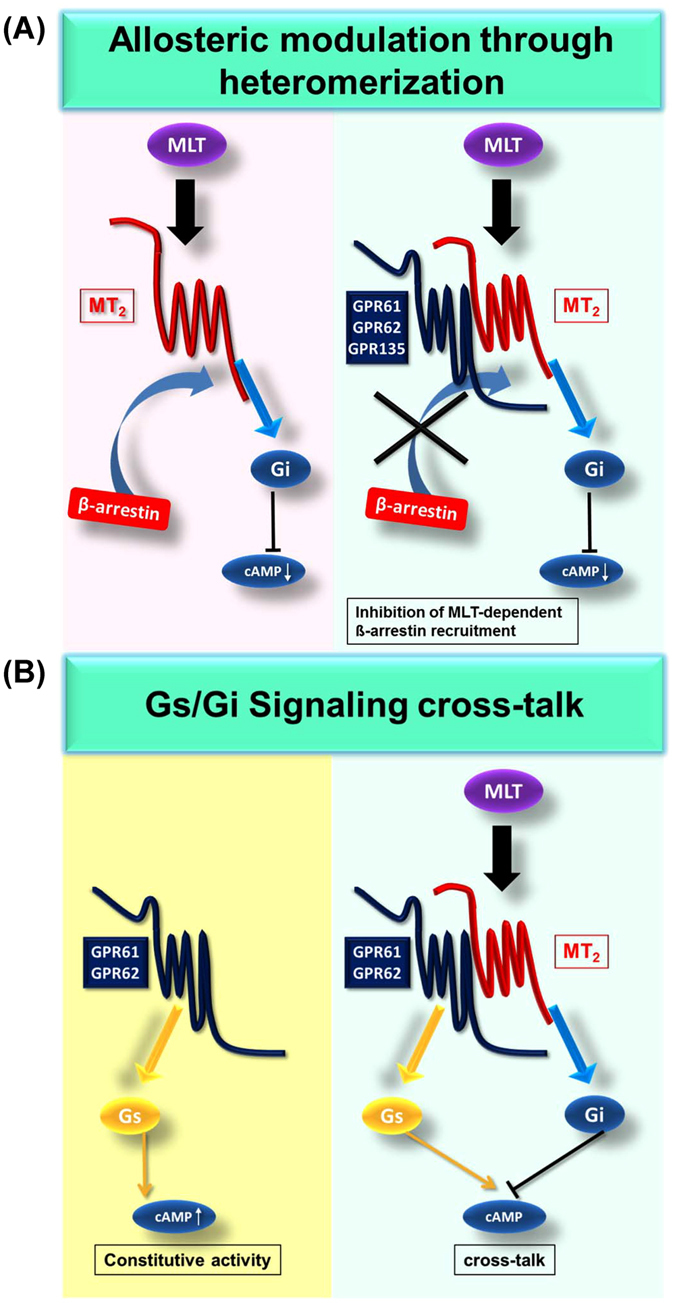
Schematic representation of the reciprocal interaction between MT_2_ receptors and orphan GPR61, GPR62 and GPR135 receptors. (**A**) Proposed model of pathway-biased allosteric modulation of MT_2_ function in heteromers with orphan GPR61, GPR62 and GPR135 receptors. In the presence of GPR61, GPR62 and GPR135, melatonin-induced ß-arrestin recruitment is decreased and G_i/o_ signaling is unaffected. (**B**) G protein signaling cross-talk between MT_2_ and GPR61 or GPR62. Constitutively active GPR61 and GPR62 stimulate cAMP production whereas melatonin stimulation of MT_2_ decreases cAMP production.

Alternatively, GPR61, GPR62 and GPR135 might also scavenge ß-arrestin away from MT_2_ due to their spontaneous ability to couple to ß-arrestin. However, this possibility seems unlikely to occur in our experimental setting since expression levels of ß-arrestin2-EA are high in the PathHunter™ enzyme complementation assay (see Fig. 3E). The absence of effect on the capacity of MT_1_ to recruit ß-arrestin2 and the AT1R-ß-arrestin2-ERK pathway further argue against this hypothesis. Collectively, we propose that GPR61, GPR62 and GPR135 are pathway-biased allosteric modulators of MT_2_ function (Fig. 8A).

The presence of an agonist-independent activity is another important feature of GPR61, GPR62 and GPR135. Whereas GPR61 showed in the current study constitutive activity on the G_s_/cAMP pathway, GPR62 showed dual constitutive activity on the G_s_/cAMP and the G_q_/IP1 pathway in HEK293T cells. The observation that GPR61 expression constitutively increases cAMP levels in HEK293T cells is in agreement with a previous study overexpressing a GPR61-G_s-alpha_ fusion protein in Sf9 cells^17^ but contrasts with another report in CHO cells suggesting that GPR61 and GPR62 expression is unable to activate a cAMP-Responsive-Element (CRE) reporter gene but rather inhibits forskolin-stimulated CRE reporter gene activity^18^. The reason for this discrepancy is currently unknown but might be explained by differences in cell types (HEK293 *vs*. CHO) or assays (cAMP production *vs*. CRE reporter gene activity). CRE mediated gene expression has indeed been shown to be sensitive to pathways other than the cAMP pathway, such as the ß-arrestin pathway^34^ or the G_q/11_/IP1 pathway^35, 36^. Such a possibility has not been ruled out by Martin *et al*.^18^.

This is the first report showing that GPR62 constitutively activates the G_q/11_/IP1 pathway. GPR61 and GPR135 were silent on this pathway. Whereas constitutive activation of G_s_/cAMP and G_i_/cAMP pathways has been reported for a number of orphan GPCRs^37, 38^, constitutive activity on the G_q/11_/IP1 pathway has been much less often observed^39, 40^.

Mutations of the conserved (D/E)RY motif of the third transmembrane domain (TM3) are well-known to be related to the constitutive activity of GPCRs^41, 42^. In addition, several orphan GPCRs with constitutive Gs activity lack the DRY motif ^37^. The glutamic acid (6.30) in the sixth transmembrane domain (TM6) is also well-known conserved amino acid residue (Supplementary Fig. S10), and is proposed to be part of the “ionic lock” which stabilizes GPCRs in the inactive state. Whereas GPR61 and GPR135 contain the (D/E)RY motif, GPR62 has a ARY motif at this position. The glutamic acid in TM6 is present in GPR135 but replaced by glycine in both GPR61 and GPR62. Although the importance of the “ionic lock” and its disruption in GPCRs other than rhodopsin is still controversial^43, 44^, the constitutive activity of GPR61 on the Gs/cAMP pathway and the dual constitutive activity of GPR62 on the Gs/cAMP and Gq/11/IP1 pathways might be explained by the absence of the ionic lock.

In addition, we show that GPR61, GPR62 and GPR135 constitutively recruit ß-arrestins. As demonstrated here and in many other studies, the amplitude of spontaneous receptor activity depends on the receptor expression level. Overexpression of receptors can thus reach spontaneous receptor activities that never occur in a physiological setting. To start to address this point we selected GPR135, which is expressed in human pancreatic beta-cells^10^, and show that spontaneous ß-arrestin recruitment can be observed at levels matching endogenous GPR135 mRNA levels observed in EndoC-βH1 cells. Spontaneous, agonist-independent ß-arrestin recruitment has been reported for other GPCRs including CCR1, mGluR1a and 5-HT_2C_ receptors^45–49^. GPR135 can be considered an interesting candidate for a ß-arrestin biased receptor in terms of its constitutive activity as no constitutive activity was detectable on the G_s_/cAMP, G_i_/cAMP and G_q/11_/IP1 pathways. Assuming that a similar activity profile is observed for endogenously expressed GPR135, this receptor might become an interesting model of a naturally ß-arrestin-biased receptor.

We show that G_i/o_ activation by MT_2_ diminishes the spontaneous activity of GPR61 and GPR62 on the G_s_/cAMP pathway but not on the G_q/11_/IP1 and ß-arrestin pathways. This is unlikely to be the result of an allosteric interaction in heteromers but rather through the signaling cross-talk between G_s_-mediated adenylyl cyclase stimulation and G_i/o_-mediated inhibition (Fig. 8B) as pertussis toxin treatment abolished the effect of MT_2_.

In conclusion, here we reported the functional analysis of GPR61, GPR62 and GPR135. They don’t bind to melatonin but are likely allosteric modulators of melatonin-induced MT_2_ function through receptor heteromerization. The second interesting feature of GPR61, GPR62 and GPR135 is their high level of agonist-independent, spontaneous activity. The constitutive activity of GPR61 and GPR62 on the G_s_/cAMP pathway is counterbalanced by the activation of the G_i/o_/cAMP pathway by MT_2_ via G protein cross-talk. Both, allosteric modulation of MT_2_ function through heteromerization and the signaling cross-talk regulation of the spontaneous activity of orphan receptors offer the possibility for tightly regulated reciprocal modulation of MT_2_ receptors and GPR61, GPR62 and GPR135 function.

## Methods

### Reagents

Melatonin was purchased from Sigma-Aldrich (St. Louis, MO, USA) and 2-[125I]iodomelatonin and [3H]melatonin from PerkinElmer (Waltham, MA, USA). cDNAs of human GPR61 (Cat. No. GPR0610000), human GPR62 (Cat. No. GPR0620000) and human GPR135 (Cat. No. HUMNP00000) were purchased from cDNA resource center (Bloomsburg, PA, USA). HA-tagged MT_2_, GPR61, GPR62 and GPR135, and 6myc-GPR61-PK2, 6myc-GPR62-PK2, and 6myc-GPR135-PK2 were constructed as previously described^50^. The DNA sequence encoding the HA epitope tag (YPYDVPDYA) were added by PCR just upstream of the MT_2_ or GPR coding regions, and PCR products were inserted to pcDNA3 (Invitrogen, CA, USA). 6myc-GPRs-PK2 fragment fusion proteins were constructed by ligating the 6myc-tag at the N-termini and PK2 fragment at the C-termini of GPR61, GPR62 and GPR135 in the pCMV-ARMS2-ProLink2 vector (DiscoverX 93–0490). For this, the coding regions of GPRs were amplified by PCR to add the EcoRI site (5′) and HindIII site (3′) and to delete stop codons of GPRs. The Flag-MT_1_ has been described previously^51^. Flag-MT_1_-PK2 and HA-MT_2_-PK2 were constructed by ligating the PK2 fragment at the C-termini of Flag-MT_1_ or HA-MT_2_ in the same way as for GPRs. All constructs were verified by DNA sequencing.

### Cell culture and transient transfection

Cell culture and transient transfection were performed as previously described^52^. Briefly, HEK293T cells were grown in complete DMEM medium, supplemented with 10% (v/v) fetal bovine serum, 4.5 g/L glucose, 100 U/ml penicillin, and 0.1 mg/ml streptomycin and maintained at 37 °C (95% O2, 5% CO2). Transient transfection of cells was performed with the JetPEI reagent according to the supplier’s instructions (Polyplus-transfection, New York, NY, USA) except ß-arrestin assay. For the ß-arrestin assay, X-tremeGENE 9 (Sigma-Aldrich) was used for transfection. Transient transfection was performed in 10 cm dish (radioligand binding assay) or 12-well plates (all the other experiments).

### Immunofluorescence

The expression of HA-tagged (N-terminus) GPR61, GPR62 or GPR135 receptors were detected by immunofluorescence as previously described^53^. Briefly HEK293T cells transiently expressing HA- or myc-tagged GPR61, GPR62 or GPR135 (1 ug DNA) were seeded onto sterile poly-L-lysine–coated 24-well glass 1 day after transfection. Next day, with or without Transferrin-Alexa555 (Molecular Probes; T-35352) treatment (5 μg/ml for 30 minuites in 37 °C after washing by serum free medium), cells were fixed with a 4% fresh paraformaldehyde solution (in PBS) for 20 min. With or without 5-min permeabilization step in 0.2% Triton X-100 (in PBS), cells were blocked for 1 hour with 5% BSA (in PBS). Polyclonal anti-HA antibody (dilution 1:500, rabbit, Cell Signaling Technology), monoclonal anti-myc antibody (dilution 1:500, 9E10 mouse, sc-40; Santa Cruz Biotechnology), or anti-ß-arrestin1/2 antibody (1:1000 dilution; D24H9 rabbit; Cell signaling) was applied followed by secondary fluorescein isothiocyanate-tagged anti-rabbit or anti-mouse antibodies (dilution 1:500, Jackson Immunotech 111-095-003). Cells were examined by confocal microscopy (Leica, Germany).

### Radioligand binding assays

Membranes from HEK293 cells transiently expressing human MT_1_, GPR61, GPR62 or GPR135 receptors were prepared as previously described^54^. 2-[^125^I]iodomelatonin and [3H]-melatonin binding experiments were performed at 300 pM and 1 nM and specific binding was defined as binding displaced by 10 µM iodomelatonin and melatonin, respectively. Assays were carried out in triplicates for 120 min at room temperature followed by rapid filtration through GF/F glass fiber filters (Whatman; Clifton, NJ, USA). Filter-retained radioactivity was determined with a gamma-counter LB2111 (Berthold Technologies; Bad Wildbad, Germany).

### cAMP assay

The cyclic AMP assay was performed as previously described^55^. Briefly, HEK293 cells expressing different quantities of HA-GPR61, HA-GPR62 or HA-GPR135 receptors were dispensed into a 384-well plate (4,000 cells per well) and stimulated with or without various forskolin concentration (described in each figure) in the presence or absence of 1 µM melatonin for 30 min at room temperature in PBS buffer supplemented with 1 mM 3-isobutyl-methylxanthine (IBMX, Sigma-Aldrich, St Quentin, France). Cells were then lysed and cAMP levels were determined following the manufacturer’s instruction (Cisbio Bioassays, Codolet, France). Perturssis toxin pretreatment was performed in 200 ng/ml for 4 hours. The plate was read using the Infinite F500 Tecan microplate reader.

### IP1 assay

IP formation was quantified with HTRF (Homogeneous Time-Resolved Fluorescence) based “Cisbio IP-One Tb” (Cisbio, Bagnols-sur-Cèze, France) assay kit as previously discribed^56^. HEK293T cell suspensions (5 × 10^4^ cells) were treated in 384-well plate (10 μl volume) with or without 1 µM melatonin in modified stimulation buffer (10 mM Hepes, 10 mM MES, 1 mM CaCl_2_, 0.5 mM MgCl_2_, 4.2 mM KCl, 146 mM NaCl, 5.5 mM glucose, 50 mM LiCl) at pH 7.4 or 6.4 for 60 min at 37 °C. IP measurements were performed in triplicates and experiments were repeated at least three times. Samples were read on a TECAN Infinite F500 (Tecan Group, Ltd., Männedorf, Switzerland) with excitation at 320 nm and emission at both 620 nm and 665 nm.

### β-arrestin2 recruitment assay

The PathHunter™ assay from the DiscoverX company (DiscoveRx, Birmingham, United Kingdom) was used to measure the recruitment of β-arrestin2 to melatonin receptors as previously discribed^57^. Briefly, HEK293 parental cells stably expressing a fusion protein of β-arrestin2 and the larger N-terminal deletion mutant of β-gal were transiently transfected by X-tremeGENE 9 with different quantitates of expression vectors for Flag-MT_1_, HA-MT_2_, myc-GPR61, myc-GPR62 or myc-GPR135 fused at their C-terminal part with the small enzyme fragment ProLink tag (PK2). On the next day, cells were spread to poly-L-lysine (Sigma)-coated 98-well plate. Two days after transfection, the cells were treated with or without 1 µM melatonin for 2–3 hours at 37 °C. β-arrestin2 recruitment resulted in the complementation of the two enzyme fragments and the formation of an active β-gal enzyme. Luminescence signals were determined after 60 min incubation at room temperature.

### SDS-Page/immunoblot analysis

Western blot was performed as previously described^58^. Briefly, denatured proteins were resolved in 12% SDS-PAGE gels, transferred to nitrocellulose membranes, and immunoblotted with antibodies against each primary antibody as bellows; anti-flag antibody (1:1000 dilution, rabbit F7425 Sigma-Aldrich), anti-HA antibody (1:1000 dilution 16B12 mouse, 901514 Biolegend), anti-myc antibody (1:1000 dilution; 9E10 mouse, sc-40; Santa Cruz Biotechnology), anti-ß-arrestin1/2 antibody (1:1000 dilution; D24H9 rabbit; Cell signaling), Immunoreactivity was revealed using secondary antibodies coupled to 680 or 800 nm fluorophores (LI-COR Biosciences, Lincoln, NE, USA), and readings were performed with the Odyssey LI-COR infrared fluorescent scanner (LICOR Biosciences).

### ELISA assay

The rest of the transfected cells used for ß-arrestin assay (Fig. 5A,C,E) were plated to a 96 well plate after 24 hours from the transfection. 48 hours later from the transfection, the cells were fixed by 50/50% methanol/acetone for 1 min at room temperature and permeabilized by 0.2% TritonX-100 for 9 minutes after several washes by PBS, the cells were blocked with 3% BSA for 1 hour at room temperature. Then cells were incubated with indicated primary antibody (1:1000 dilution anti-HA; 16B12 mouse 901514 Biolegend, 1:1000 dilution anti-Flag; F3165 mouse SIGMA, 1:10000 dilution anti-GFP; rabbit ab290 abcam) in 3% BSA for overnight at 4 °C. After washing the plate 4 times with PBS, plate was incubated with 50 µL/well of secondary antibody (1:20000 dilution; HRP conjugated anti-mouse IgG DC02L Millipore, 1:20000 dilution; HRP conjugated anti-rabbit antibody A0545 Sigma) for 1 hour at room temperature. After washing the plate 6 times with PBS, 50 µL/well of LuminataTM Crescendo ELISA HRP Substrate (Millipore) were added and the luminescence were read by TECAN Infinite F500 (Tecan Group, Ltd., Männedorf, Switzerland).

### Bioluminescence Resonance Energy Transfer (BRET) Measurement

For BRET donor saturation curves were obtained as previously described^59^, HEK293 cells seeded in 24-well plates were transiently transfected with 3 ng of GPR61-Rluc, 0.8 ng of GPR62-Rluc, 0.2–0.4 ng of GPR135-Rluc and 5–400 ng of the corresponding YFP plasmids. 24 h after transfection, cells were transferred into a 96-well white Optiplate (Perkin Elmer Life Sciences) precoated with 10 μg/ml poly-L-lysine (Sigma) and incubated for another 24 h before BRET measurements. Luminescence and fluorescence were measured simultaneously using the TECAN Infinite F500 (Tecan Group, Ltd., Männedorf, Switzerland).

### Co-immunoprecipitation assay

MT_2_-YFP (900 ng of DNA per 0.3 × 10^6^ cells/12 well dish) with or without HA-GPR61, HA-GPR62 or HA-GPR135 (100 ng of DNA per 0.3 × 10^6^ cells/12 well dish) were transfected into HEK293T cells. After a further 48 h, the cells were lysed in immunoprecipitation buffer (50 mM Tris-HCl, pH7.5, 150 mMNaCl, 10 mMEGTA, 1 mM DTT, 1% Triton-X 100, and proteinase inhibitors) for 1 h at 4 °C. The lysates were incubated with 0.4 μg/sample of rat monoclonal anti-HA antibody (Sigma, 3F10) for 1 h at 4 °C with tumble. This was followed by the addition of 20 μl of ProteinG solution (Sigma, 50% in resin bed) and tumbled for another 1 h at 4 °C. After several further washes with immunoprecipitation buffer, the resins were vortexed and heated at 55 °C for 15 min after adding equal volume of 2xLaemmli sample buffer. The proteins were separated by SDS-PAGE and Western blot analysis was performed using polyclonal anti-GFP (1:2000 dilution, rabbit, ab6556 Abcam) or anti-HA (1:2000 dilution 16B12 mouse, 901514 Biolegend) and the indicated secondary antibody for each.

### Statistical analysis

Results were presented as mean ± standard error of the mean (SEM). Statistical significance was assessed with two tailed t-test (Fig. 3) and one-way ANOVA (Figs 1 and 5) or two-way ANOVA (Fig. 7) followed by appropriate post-hoc test. P-values less than 0.05 (P < 0.05) were considered statistically significant. The analysis was done by using Graph-pad Prism.

## Electronic supplementary material

Supplementary Figures
