# Supplementary material for: Orphan GPR61, GPR62 and GPR135 receptors and the melatonin MT2 receptor reciprocally modulate their signaling functions
Source: Sci Rep. 2017 Aug 21;7:8990. doi: 10.1038/s41598-017-08996-7 (PMC5566548; doi:10.1038/s41598-017-08996-7)
Supplement: Supplementary file 1 — Supplementary Figures [file 41598_2017_8996_MOESM1_ESM.pdf]

Supplementary Information

Orphan GPR61, GPR62 and GPR135 receptors and the melatonin MT_2 receptor reciprocally modulate their signaling functions

Atsuro Oishi, Angeliki Karamitri, Romain Gerbier, Olivier Lahuna, Raise Ahmad and Ralf Jockers*

Supplementary Figure S1.

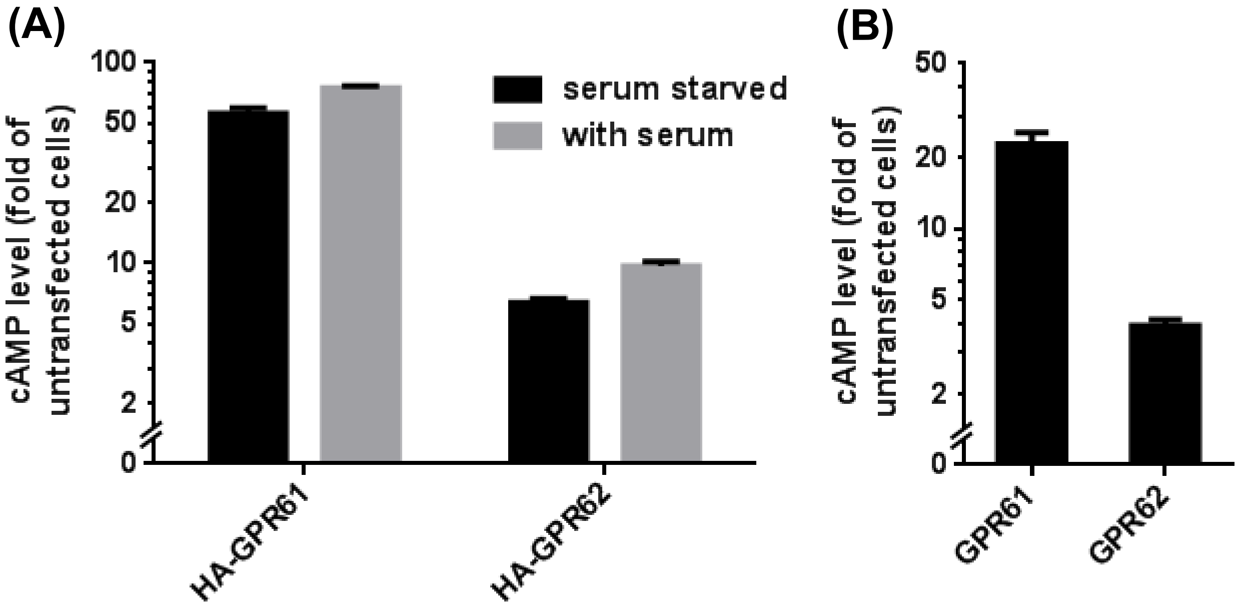

Supplementary Fig. S1. (A) The effect of serum on basal cAMP accumulation in HEK293T cells expressing HA-GPR61, HA-GPR62. HEK293T cells transfected 1mg vector DNA of HA-GPR61, HA-GPR62 were serum starved for 16 hours and majored cAMP acumulation. **(B) Basal cAMP accumulation in HEK293T cells expressing GPR61, GPR62 without any tag.** HEK293T cells transfected 1mg vector DNA of GPR61, GPR62 were majored cAMP acumulation. Data represent the mean $\pm$ S.E.M of at least three independent experiments performed in triplicates.

Supplementary Figure S2.

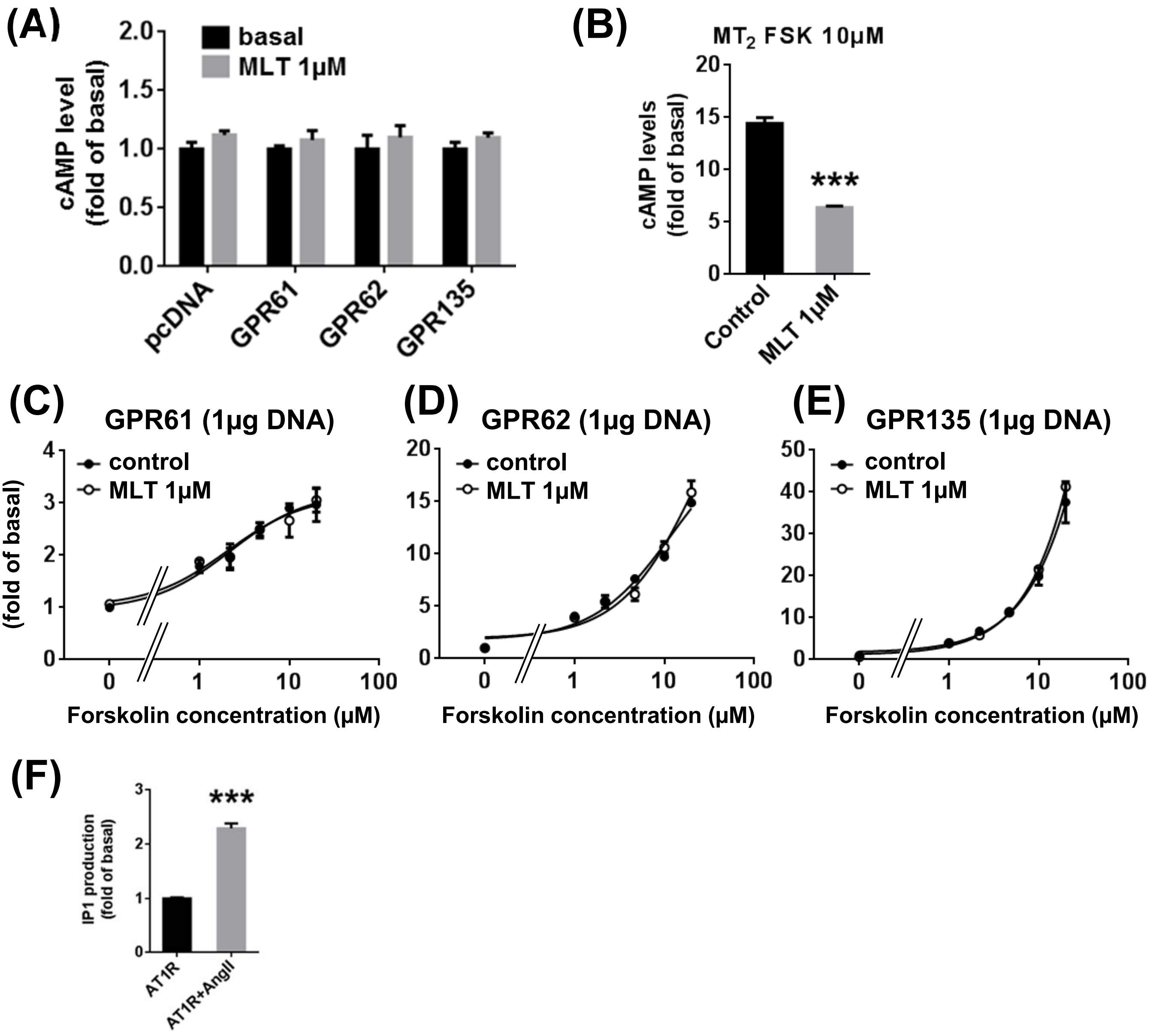

Supplementary Fig. S2. The effect of Melatonin on cAMP levels through GPR61, GPR62 and GPR135. Effect of melatonin (MLT) (1μM) on basal **(A)** and forskolin-stimuated **(B-E)** cAMP levels in HEK293T cells transfected with 1μg of HA-MT_2, HA-GPR61, HA-GPR62 or HA-GPR135 expression vector. **The effect of Angiotensin II on IP1 through AT1R. (F)** Angiotensin II-induced IP1 production in cells expressing angiotensin II type I receptor was used as positive control. Data are expressed as mean ± SEM. from three independent experiments performed in triplicates. ***, $P < 0.0001$

Supplementary Figure S3.

(A)

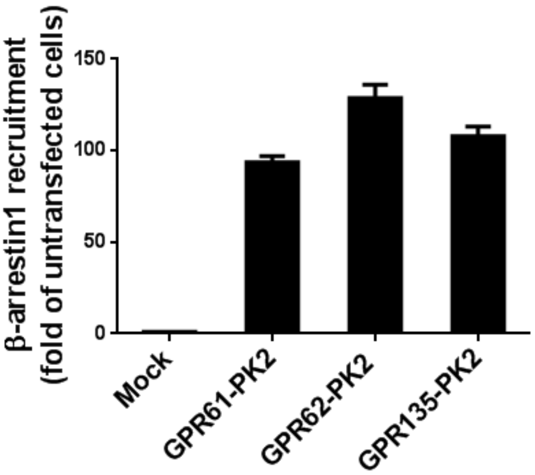

(B)

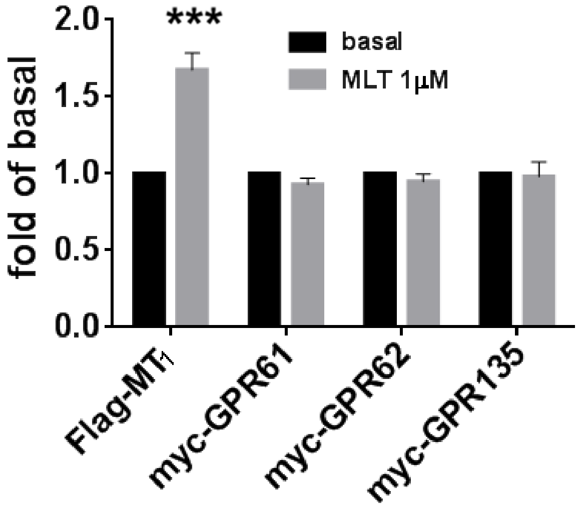

Supplementary Fig. S3. (A) The agonist independent ß-arrestin1 recruitment of GPR61, GPR62 and GPR135. ß-arrestin recruitment was determined in HEK-arrestin1-EA cells stably expressing the ß-arrestin1-EA fusion protein and transfected with 1μg of myc-GPR61-PK2, myc-GPR62-PK2 or myc-GPR135-PK2 compared with untransfected cell. **(B) MLT induced ß-arrestin1 recruitment of GPR61, GPR62 and GPR135.** Effect of melatonin (1μM) on ß-arrestin1 recruitment to GPR61, GPR62 and GPR135 (1ug). Flag-MT1-PK2 was used as a positive control. Data represent the mean ± S.E.M of at least three independent experiments performed in triplicates.

Supplementary Figure S4.

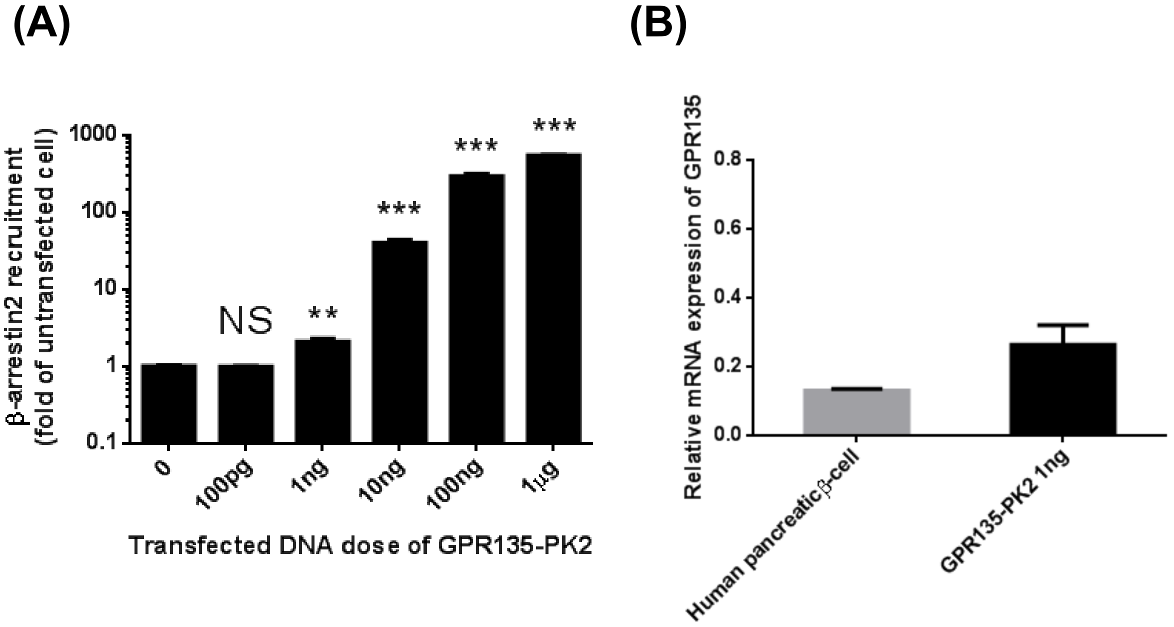

Supplementary Fig. S4. ß-arrestin2 recruitment at different amounts of transfected GPR135-PK2 expression vector (A) and mRNA level of endogenously expressed GPR135 (B). (A) ß-arrestin recruitment was determined in HEK-arrestin2-EA cells stably expressing the ß-arrestin2-EA fusion protein and transfected with different amounts of myc-GPR135-PK2 expression vector. (B) cDNAs were obtained from human pancreatic ß-cell and HEK-arrestin2-EA with transfection of 1ng of myc-GPR135-PK2. mRNA of GPR135 and HPRT1 was determined by real-time PCR with TaqMan® Gene Expression Assays (Thermofisher) for human GPR135 (Hs00254758_s1) and human HPRT1 (Hs02800695_m1). Relative mRNA expression of GPR135 was normalized with mRNA expression of HPRT1. Data are expressed as mean ± SEM from 3 independent experiments performed in duplicates. **, $P < 0.01$, ***, $P < 0.001$

Supplementary Figure S5.

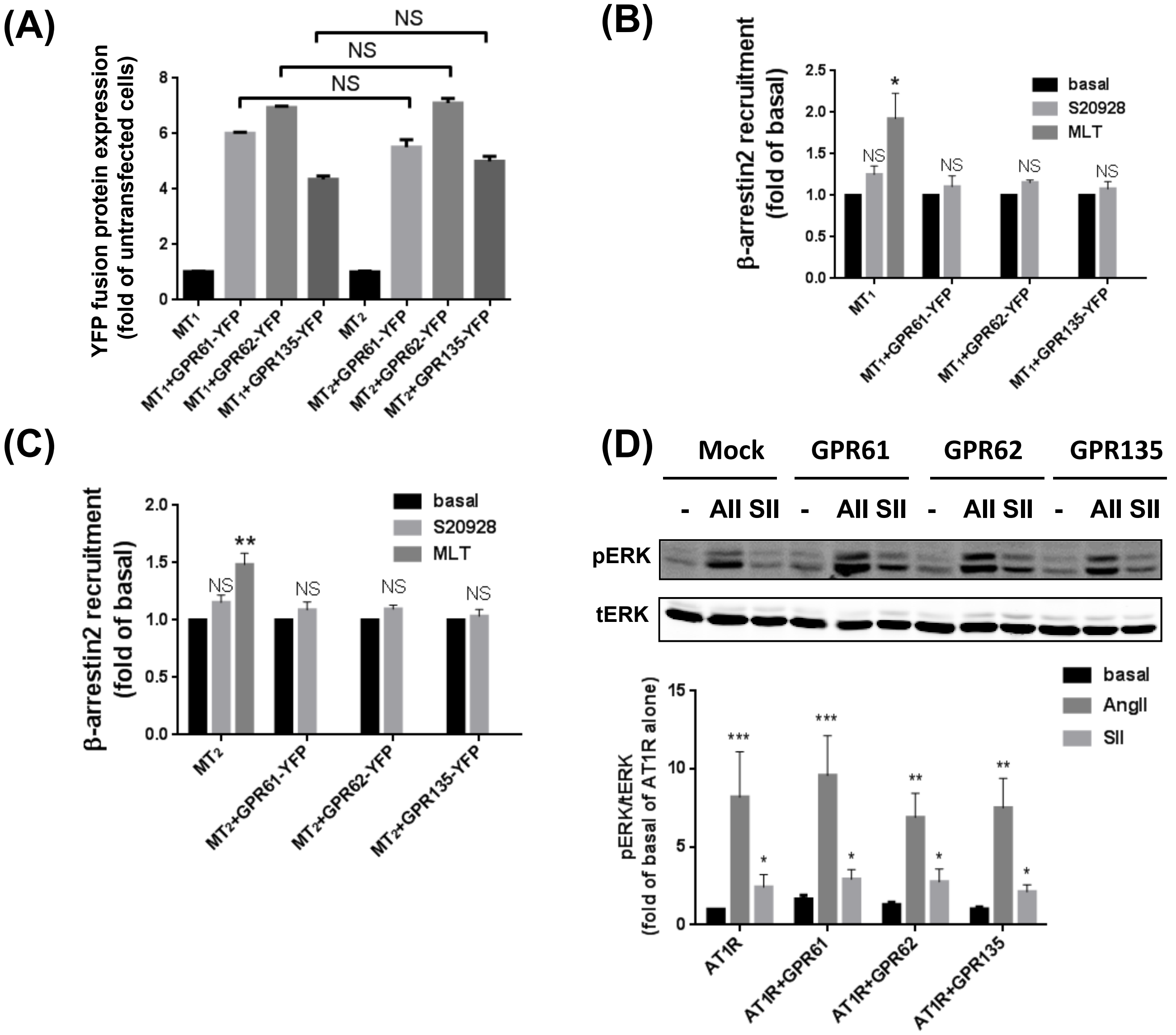

Supplementary Fig. S5. Expression level of GPR61, GPR62 and GPR135 co-expressed with MT_1 or MT_2 in the melatonin induced β-arrestin2 recruitment assay. (A) Expression levels of GPR61-YFP, GPR62-YFP or GPR135-YFP co-expressed with MT_1 (shown in Fig.5A,B) or MT_2 (shown in Fig.5C,D) monitored by ELISA. Expression level of each GPR were not significantly different in cells co-expressing MT_1 or MT_2. **Effect of the melatonin receptor antagonist S20928 on β-arrestin2 recruitment to MT_1 and MT_2 in the absence and presence of GPR61, GPR62 and GPR135 (B-C)** The effect of S20928-induced ß-arrestin recruitment was determined in HEK-arrestin-EA cells stably expressing the ß-arrestin2-EA fusion protein and transfected with Flag-MT1-PK2 (B), HA-MT2-PK2 (C) in the absence or presence of GPR61-YFP, GPR62-YFP or GPR135-YFP. In every condition, S20928 didn't increase ß-arrestin2 recruitment significantly. **Effect of GPR61, GPR62 and GPR135 on Angiotensin II or [Sar^1-Ile^4-Ile^8]-Angitotensin II (SII) induced phosphorylation of ERK1/2 through AT1R.(E)** The expression of GPR61, GPR62 and GPR135 didn't change phosphorylation of ERK1/2 through AT1R significantly.

Supplementary Figure S6.

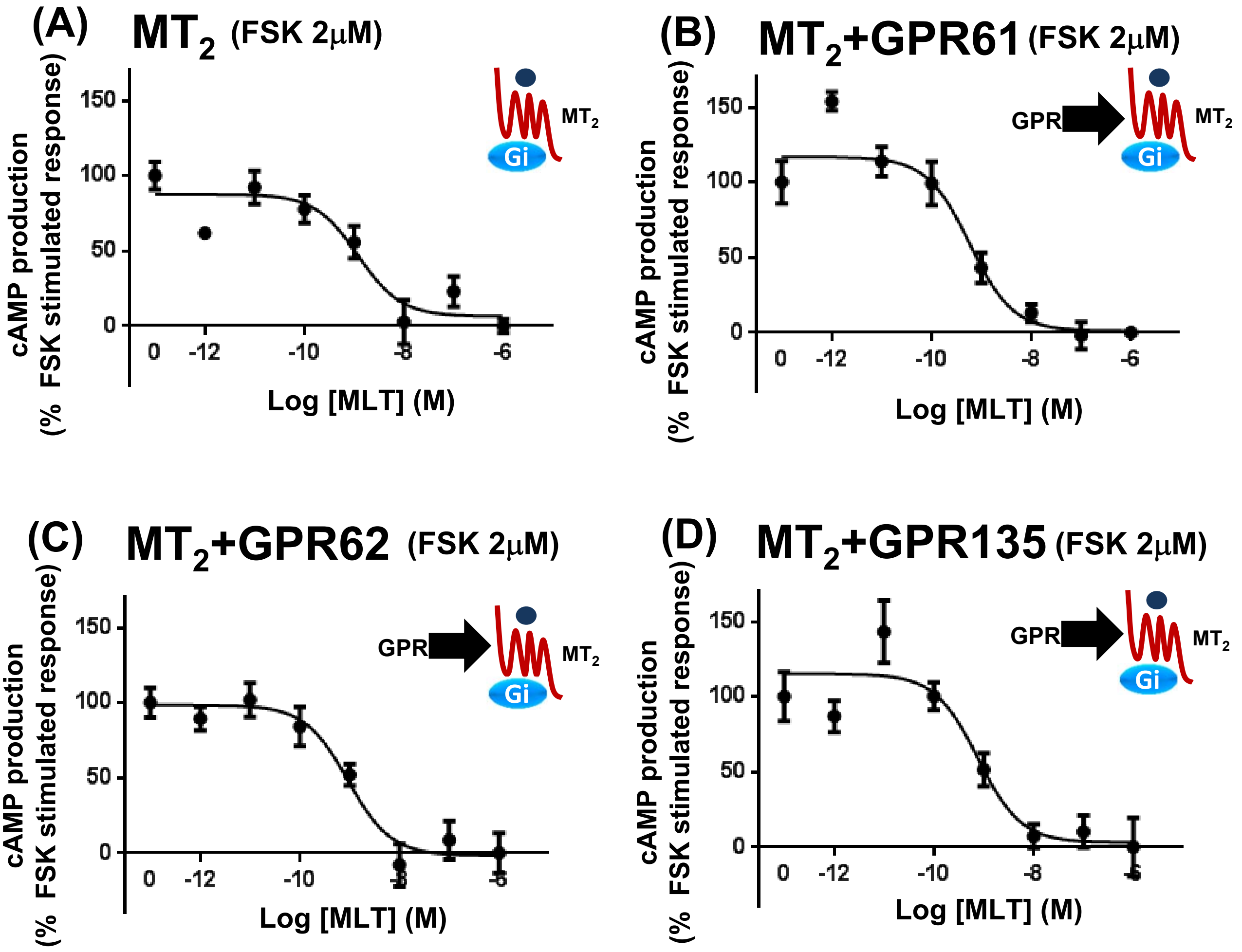

(E)

	pEC_{50}
MT_2	8.92 ± 0.25
MT_2+GPR61	9.23 ± 0.18
MT_2+GPR62	9.03 ± 0.22
MT_2+GPR135	9.12 ±0.26

Supplementary Fig. S6. Effect of GPR61, GPR62 and GPR135 on MT_2 promoted inhibition of forskolin induced cAMP production. HEK-arrestin-EA cells expressing MT_2 alone **(A)**, or together with GPR61 **(B)**, GPR62 **(C)** or GPR135 **(D)** were treated with 2 μM forskolin (FSK) and increasing concentrations of melatonin (MLT). **(E)** Melatonin potency was expressed as pEC_{50} derived from GraphPad Prism. Data represent the mean ± S.E.M of at least three independent experiments performed in triplicates.

Supplementary Figure S7.

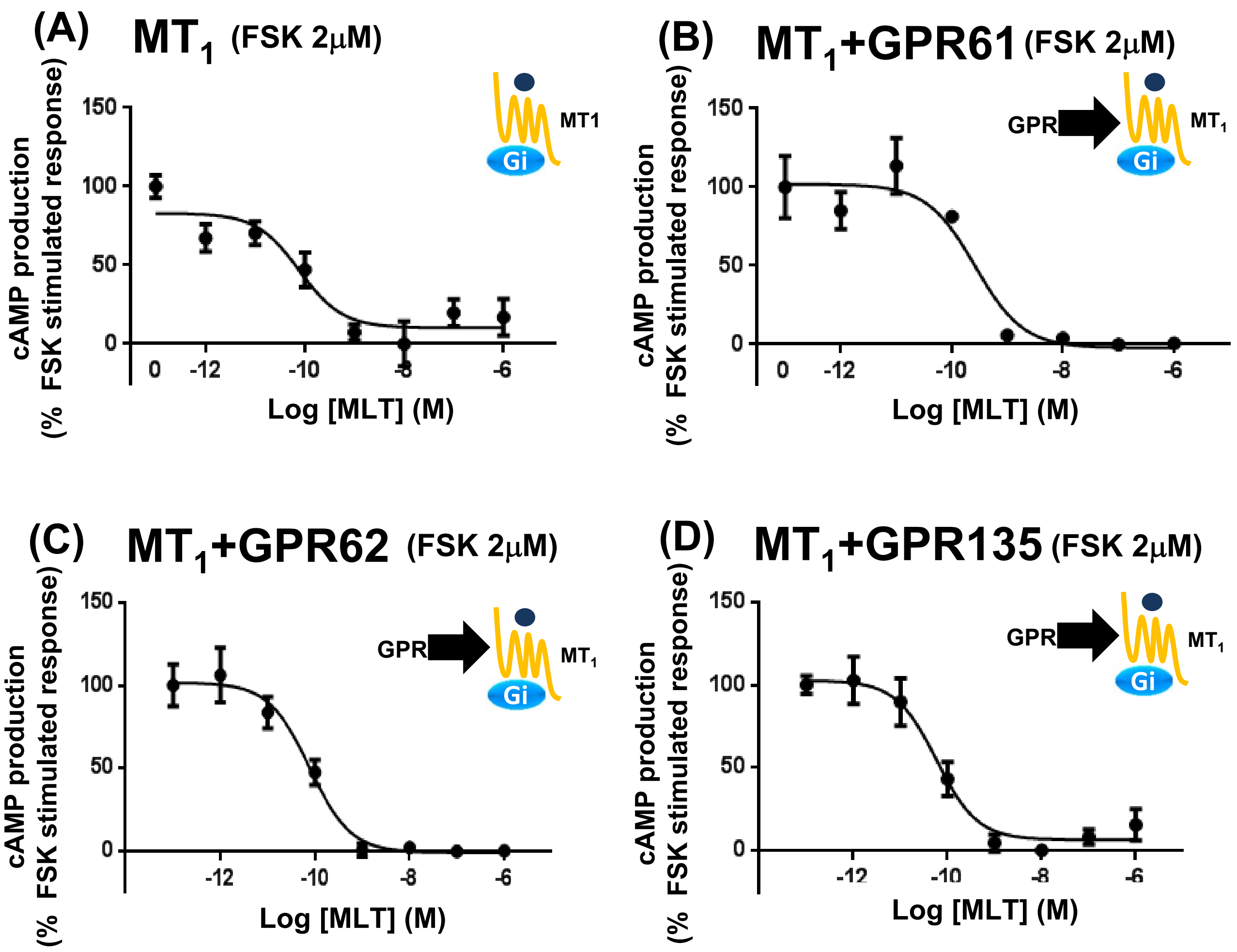

(E)

	pEC_{50}
**MT_1**	**10.11 ± 0.28**
**MT_1+GPR61**	**9.58 ± 0.21**
**MT_1+GPR62**	**10.11 ± 0.15**
**MT_1+GPR135**	**10.23±0.18**

Supplementary Fig.S7. Effect of GPR61, GPR62 and GPR135 on MT_1 promoted inhibition of forskolin induced cAMP production. HEK-arrestin-EA cells expressing MT_1 alone **(A)**, or together with GPR61 **(B)**, GPR62 **(C)** or GPR135 **(D)** were treated with 2 μM forskolin (FSK) and increasing concentrations of melatonin (MLT). **(E)** Melatonin potency was expressed as pEC_{50} derived from GraphPad Prism. Data represent the mean ± S.E.M of at least three independent experiments performed in triplicates.

Supplementary Figure S8.

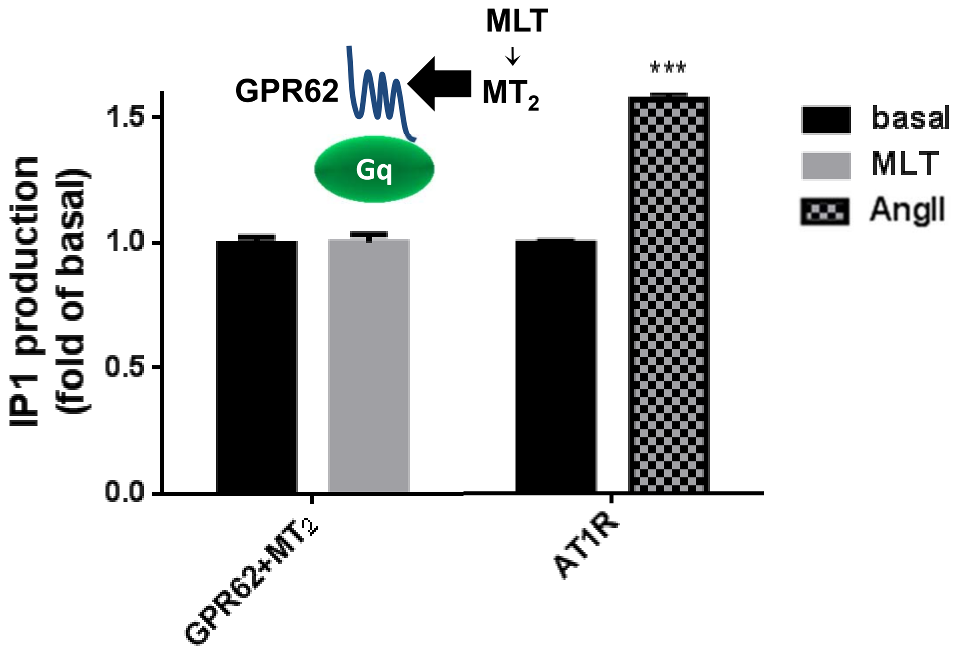

Supplementary Fig. S8. Effect of melatonin through MT2 on GPR62 promoted IP1 production. Inositol phosphate (IP1) production was determined in HEK293T cells in 12 well plates transfected with 500ng of HA-GPR62 and 500ng of MT2-YFP expression vectors in the absence or presence of 1µM melatonin (MLT). Angiotensin II-induced IP1 production in cells expressing angiotensin II type I receptor was used as positive control. Data are expressed as mean ±SEM. from three independent experiments performed in triplicates. ***, P < 0.001

Supplementary Figure S9.

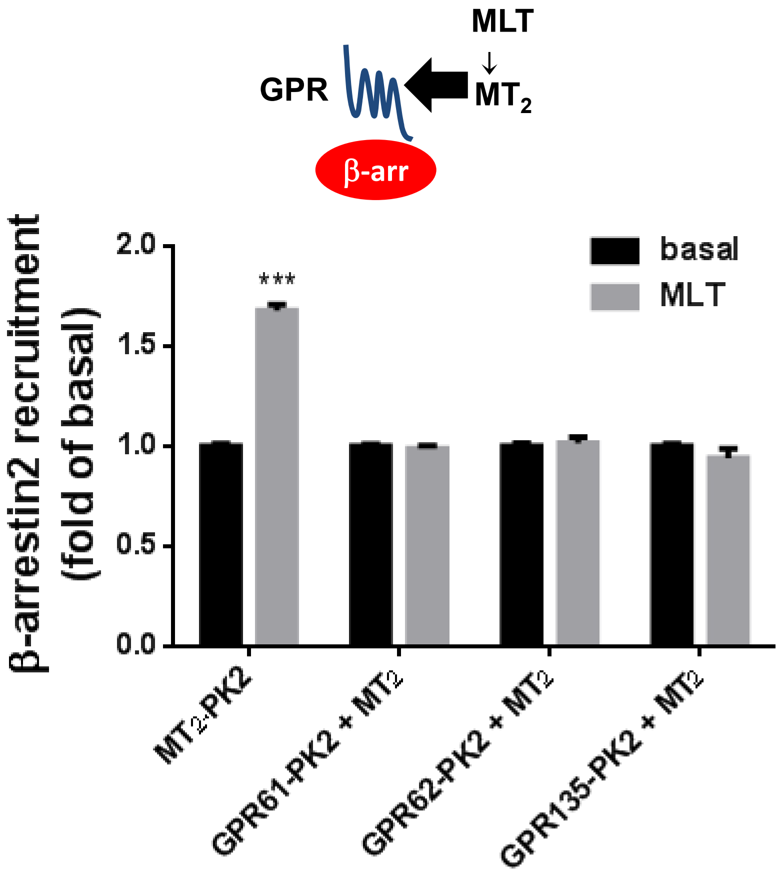

Supplementary Fig. S9. Effect of melatonin through MT2 on spontaneous association between GPR61/GPR62/GPR135 and ß-arrestin2. Effect of melatonin (1μM) was examined on ß-arrestin2 recruitment to GPR61-PK2, GPR62-PK2 and GPR135PK2 (500ng) in the presence of HA-MT2 (without PK2-fragment, 500ng). HA-MT2-PK2 was used as a positive control. Data are expressed as mean ± SEM from 3 independent experiments performed in triplicates. ***, $P < 0.001$

Supplementary Figure S10.

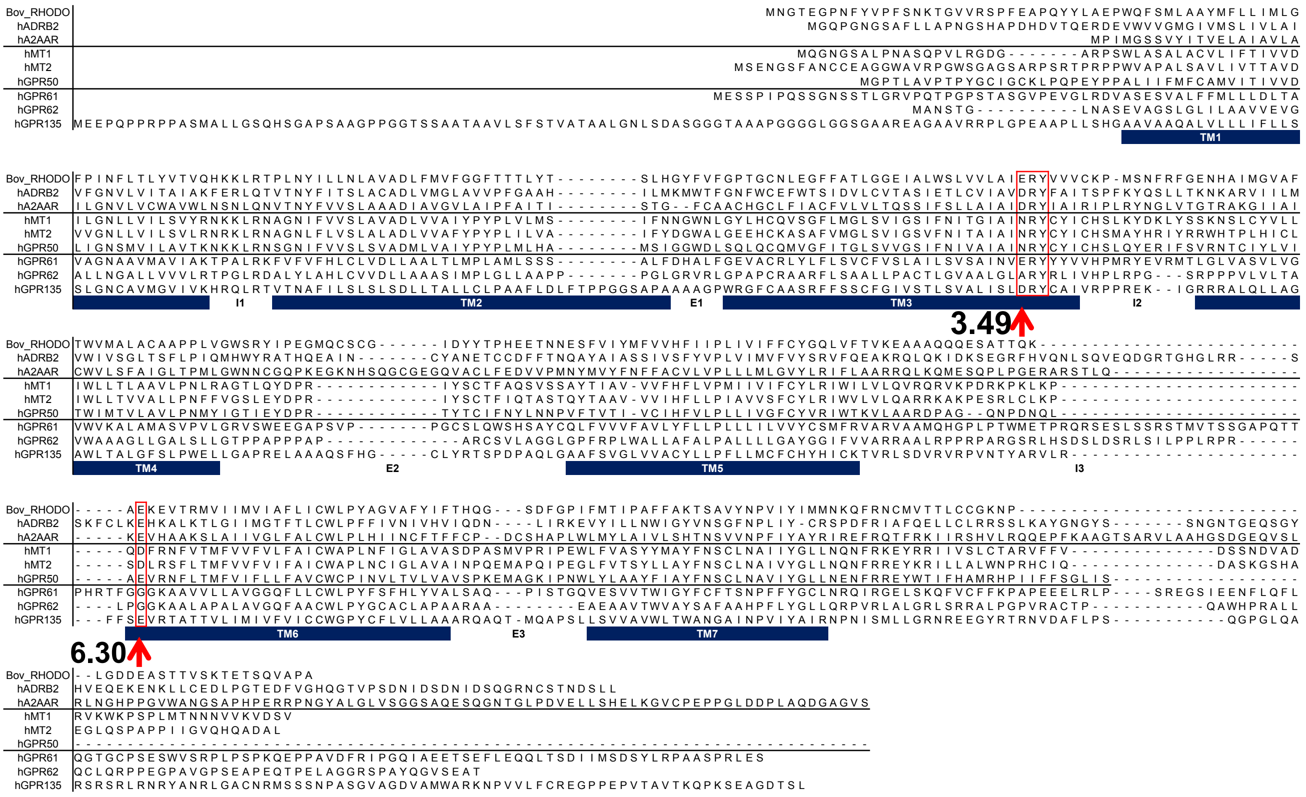

Supplementary Fig. S10. Sequence alignment of known and suspected melatonin receptor subfamily members. Alignment of amino acid sequences of established (human MT1, MT2 and GPR50) and suspected (GPR61, GPR62 and GPR135) members of the melatonin receptor subfamily together with GPCRs with high sequence homology to this subfamily and solved crystal structure (bovine rhodopsin, human ß2-adrenergic receptor, human adenosine A2 receptor). Predicted transmembrane (TM) regions and intracellular (I) and extracellular (E) loops are indicated. Particular amino acid motifs (see text) are highlighted in boxes. Amino acids beyond position 298 of GPR50 were omitted for clarity. Genbank accession numbers are: MT1; **NM005958**, MT2; **AY521019**, GPR50; **NM_004224**, GPR61; **NM_031936**, GPR62; **NM_080865**, GPR135; **NM_022571**.
